# Quantitative Photoacoustic Vascular and Hemodynamic Imaging: Complementary Assessment with Reference Modalities

**DOI:** 10.3390/bios16100543

**Published:** 2026-09-28

**Authors:** Ninjbadgar Tsedendamba, Jaeeun Seo, Wonseok Choi

**Affiliations:** 1 Department of Biomedical Engineering and Medical Sciences, College of Medicine, The Catholic University of Korea, Seoul 06591, Republic of Korea; ninjbadgar@catholic.ac.kr; 2 Major in Applied Artificial Intelligence, Graduate School of Convergence Science and Technology, Pohang University of Science and Technology, Pohang 37673, Republic of Korea; jaeeun@postech.ac.kr

**Keywords:** photoacoustic imaging, functional imaging, blood oxygenation, vascular imaging

## Abstract

Photoacoustic imaging (PAI) provides label-free visualization of vascular structure and tissue oxygenation by combining optical absorption contrast with acoustic detection, offering a favorable depth-to-resolution trade-off compared with conventional optical techniques. However, quantitative interpretation of PAI-derived vascular and hemodynamic biomarkers remains challenging because of heterogeneous optical fluence, motion, sequential wavelength acquisition, and limited standardization across imaging platforms and analysis methods. This review summarizes studies that assess PAI-derived vascular and hemodynamic parameters alongside established reference modalities, including near-infrared spectroscopy, photoplethysmography, laser speckle contrast imaging, hyperspectral imaging, Doppler and ultrafast ultrasound, ultrasound localization microscopy, and magnetic resonance imaging. Particular emphasis is placed on the inter-modality relevance of vascular and hemodynamic biomarkers, categorized as direct, indirect, or complementary assessment, across measurements of vascular morphology, perfusion, blood-flow dynamics, hemoglobin-related changes, and oxygenation. Across these studies, reference modalities provide structural or physiological context for interpreting PAI measurements, while PAI offers spatially and depth-resolved assessment of vascular and oxygenation heterogeneity. A better understanding of these inter-modality relationships, combined with standardized quantitative analysis, will be important for improving the reliability, physiological interpretation, and clinical translation of PAI-derived vascular and hemodynamic biomarkers.

## 1. Introduction

Biomedical photoacoustic imaging (PAI) is a hybrid optical-acoustic imaging modality that has been extensively investigated for label-free visualization and functional characterization of endogenous chromophores in living tissues [1,2,3,4]. In principle, PAI can selectively visualize the distribution of tissue chromophores according to their wavelength-dependent optical absorption properties. Among endogenous chromophores, oxyhemoglobin (*HbO*_2_) and deoxyhemoglobin (*HbR*) are particularly important biomarkers of vascular function and tissue oxygenation [5,6,7,8]. By detecting optical absorption-induced acoustic waves (i.e., the photoacoustic (PA) effect), PAI can visualize hemoglobin-containing blood vessels and perfused tissues at depths up to several centimeters while maintaining sub-millimeter spatial resolution, without requiring exogenous contrast agents [9,10]. Broadly, PAI can be implemented in three domains depending on the signal generation and detection schemes: (1) PA microscopy (PAM): optical-resolution (OR) or acoustic-resolution (AR); (2) PA mesoscopy (PAMes); and (3) PA tomography (PAT) [11,12,13,14]. PAM techniques are designed for nano- to micro-scale targets (e.g., cells, organoids, capillaries, etc.) [15], PAMes for micro- to meso-scale targets at intermediate imaging depths (e.g., microvascular networks and small tissue structures) [16], whereas PAT is used for beyond micro- to milli-scale targets (e.g., whole organs, animals, and humans) [17,18,19].

Compared with conventional vascular and functional imaging modalities, including computed tomography (CT) angiography, magnetic resonance imaging (MRI), and Doppler ultrasound (US), PAI offers the distinct capability of providing label-free, hemoglobin-specific information on tissue oxygenation and vascular function without ionizing radiation or exogenous contrast agents [20]. Other optical modalities, such as photoplethysmography (PPG), near-infrared spectroscopy (NIRS), laser speckle contrast imaging (LSCI), and hyperspectral imaging (HSI), also exploit the strong optical absorption of hemoglobin. However, PAI offers a favorable depth-to-resolution trade-off for imaging subsurface tissues by combining optical excitation with acoustic detection [21,22,23].

PAI has been investigated for a broad range of diagnostic applications involving hemoglobin-based biomarkers, with the potential to improve disease detection, characterization, and monitoring. For example, cancer and inflammatory diseases may be characterized by abnormal vascularization and altered oxygen saturation (*sO*_2_) [24,25,26,27], whereas ischemic vascular diseases may exhibit impaired oxygenation and abnormal perfusion responses to physiological challenges or therapeutic interventions [28,29]. The non-ionizing and label-free nature of PAI also enables repeated measurements for longitudinal monitoring, provided that laser exposure remains within applicable safety limits [30]. These capabilities have stimulated growing interest in the quantitative assessment of vascular and hemodynamic parameters using multispectral PAI [31].

Despite these advantages, the quantitative reliability and physiological interpretation of PA-derived vascular and hemodynamic biomarkers remain important challenges [32,33]. In particular, the heterogeneous optical fluence distribution within tissue can substantially affect the quantitative accuracy of multispectral PA measurements and consequently bias estimates of hemoglobin concentration and *sO*_2_. Motion and physiological variability can further complicate multispectral measurements, particularly when sequential wavelength acquisition is required [34]. Although recent advances in high-repetition-rate laser sources and imaging hardware have improved temporal sampling [35], acquisition speed remains an important consideration for dynamic and deep-tissue imaging. Furthermore, PAI systems and reconstruction or quantitative analysis algorithms remain highly diverse, with many configurations being customized for specific research applications and limited standardization across platforms [36,37,38]. These factors make independent assessment of PA-derived vascular and functional biomarkers particularly important for establishing their physiological relevance, quantitative reliability, and reproducibility in vivo.

In this context, this review summarizes studies that have investigated PA-derived vascular and hemodynamic biomarkers alongside established imaging modalities used for vascular, perfusion, or oxygenation assessment (Figure 1). This work excludes PAI studies using exogenous contrast agents to strictly focus on vascular imaging and hemodynamics based on endogenous hemoglobin. Relevant studies were identified through targeted literature searches and reference screening, primarily using Google Scholar and Web of Science, with an emphasis on studies comparing or jointly applying PAI with independent modalities for the assessment of vascular structure, perfusion, blood flow, or oxygenation. In particular, we focused on in vivo studies that performed inter-modality assessments of PAI and reference modalities on the same imaging target within the same field of view. We organize these studies according to the reference modality, including MRI, optical sensing and imaging techniques, and US, and examine the quantitative metrics, experimental protocols, and relationships between PA-derived and reference measurements. To facilitate the maturation and broader clinical adoption of PAI, rigorous comparison with established reference modalities will be essential for establishing the reliability, physiological significance, and clinical utility of PA-derived quantitative biomarkers.

## 2. Vascular and Hemodynamic Biomarkers in Reference Modalities

### 2.1. Near-Infrared Spectroscopy (NIRS)

NIRS is an optical sensing technique that quantifies tissue oxygenation and hemoglobin-related hemodynamics by measuring the wavelength-dependent attenuation of near-infrared (NIR) light propagating through biological tissues [44,45]. Because *HbO*_2_ and *HbR* have distinct absorption spectra in the NIR range, multi-wavelength measurements can be used to estimate their relative concentrations, total hemoglobin (*HbT* = *HbO*_2_ + *HbR*), and tissue oxygen saturation (*StO*_2_). NIRS is widely used for monitoring cerebral and peripheral tissue oxygenation, but its measurements represent a relatively large tissue volume and are strongly influenced by optical scattering [46,47]. Thus, NIRS provides decent temporal monitoring of regional oxygenation but limited spatial resolution compared with imaging-based modalities.

### 2.2. Photoplethysmography (PPG)

PPG is another optical sensing technique that detects pulsatile changes in blood volume by measuring light intensity variations through or reflected from tissue. Typically, one or more light-emitting diodes with different wavelengths illuminate the tissue while a photodetector records the changes in optical intensity caused by cardiac-driven fluctuations in arterial blood volume. A PPG signal can be separated according to the rate of signal variation: (1) alternating current (AC) component reflects pulsatile arterial blood-volume changes; (2) direct current (DC) component represents the relatively static optical absorption and scattering from the background tissue or slow venous flows. PPG can therefore provide parameters such as heart rate, pulse amplitude, pulse transit or arrival time, and peripheral oxygen saturation (*SpO*_2_) [48,49,50]. However, similar to NIRS, PPG measurements are made in relatively superficial tissues (usually measured at the fingertip) and cannot directly resolve individual vessels. PPG is particularly useful as a temporal physiological reference for pulsatile hemodynamics and oxygenation rather than high-resolution vascular differentiation.

### 2.3. Hyperspectral Imaging (HSI)

HSI is an optical imaging modality that acquires spatially resolved reflectance or fluorescence spectra over a broad range of wavelengths, enabling the characterization of tissue composition and physiological states. For vascular and hemodynamic assessment, the distinct absorption spectra of *HbO*_2_ and *HbR* can be exploited to estimate superficial tissue or vascular *sO*_2_, depending on the acquisition and calibration approaches [51,52,53]. Similarly, HSI can also provide spectral indices related to pigmentation, water content, and other endogenous or exogenous chromophores [54,55]. Because HSI relies on diffuse optical measurements, it generally provides wide-field imaging with high surface sensitivity but limited penetration depth.

### 2.4. Laser Speckle Contrast Imaging (LSCI)

LSCI is another optical imaging modality that provides wide-field mapping of superficial tissue perfusion by analyzing the fluctuations in laser speckle patterns generated by moving cells. When a coherent laser beam illuminates tissue, moving erythrocytes induce temporal fluctuations in the detected speckle pattern and result in speckle blurring during camera integration, quantified as the speckle contrast K = σ/μ, where σ and μ are the standard deviation and mean of speckle intensity, respectively. Lower speckle contrast corresponds to faster blood-cell motion, allowing calculation of a relative blood flow or perfusion index [56]. LSCI can provide high-frame-rate, wide-field visualization of microvascular perfusion without exogenous contrast agents and is therefore widely used for monitoring cerebral and cutaneous microcirculation. Conventional LSCI primarily provides relative rather than absolute flow measurements, and the quantitative relationship between speckle contrast and blood flow depends on exposure time, static scattering, vessel geometry, and tissue optical properties [57,58,59].

### 2.5. Doppler US

Doppler US serves as a clinically established reference imaging modality for vascular flow and structure. Doppler US quantifies blood flow velocity or power by detecting frequency or phase shifts in US echoes from moving blood cells [60,61,62]. The Doppler frequency shift is related to the velocity component of blood flow along the US beam, allowing estimation of parameters related to blood flow velocity and flow direction. Conventional spectral and color Doppler provide velocity information predominantly in larger vessels, whereas power Doppler provides vascular structural information from improved sensitivity to low-flow signals. More recently, ultrafast Doppler (UFD) approaches based on high-frame-rate plane-wave imaging and spatiotemporal clutter filtering enable improved visualization of slow microvascular flow and estimation of vascular density or perfusion-related indices [63]. Doppler US measurements depend on the angle between the US beam and blood flow direction and are highly sensitive to tissue motion, requiring appropriate compensation for such effects.

### 2.6. Ultrasound Localization Microscopy (ULM)

ULM is a super-resolution vascular imaging modality based on contrast-enhanced US by localizing and tracking individual or sparse microbubbles with sub-diffraction precision [64,65]. After systemic administration of microbubble contrast agents, fast US imaging sequences are acquired, and individual microbubbles are detected, localized, and tracked over time. Accumulating these trajectories generates super-resolved maps of the microvascular network, while the displacement of tracked microbubbles in each frame acquisition interval translates into quantitative measurements of blood flow velocity and direction [66,67]. ULM can characterize parameters including microvascular density, vessel diameter, vessel connectivity, and flow velocity, with spatially resolving vessels beyond the conventional acoustic diffraction limit. Unlike conventional Doppler US, however, ULM requires an exogenous microbubble contrast agent and relatively long acquisitions to obtain sufficient microbubble trajectories.

### 2.7. Magnetic Resonance Imaging (MRI)

MRI provides vascular and hemodynamic measurements through tissue-specific magnetic resonance contrast mechanisms and flow-sensitive pulse sequences. Time-of-flight (TOF) and phase-contrast MRI can visualize vessels and quantify blood flow velocity [68,69,70]. Especially, functional MRI based on the blood-oxygen-level-dependent (BOLD) effect detects changes in *HbR*-related magnetic susceptibility and is therefore sensitive to changes in oxygenation and vascular activity [71,72]. Although BOLD is an indirect measurement of tissue oxygenation rather than a direct measurement of *sO*_2_, its major advantages of deep tissue penetration and whole-organ coverage provide a reliable reference for both anatomical and functional measurements.

Figure 2 summarizes the corresponding biomarkers, imaging or sensing depth, resolution, quantitative measure, and inter-modality relevance. Particularly, we focus on the inter-modality relevance of PA-derived biomarkers and physiologically related measurements obtained from reference modalities. Direct assessment refers to the cross-modal evaluation of the same or directly corresponding parameters across modalities, whereas indirect assessment involves physiologically related but non-equivalent biomarkers or responses. Complementary assessment refers to the combined interpretation of distinct vascular or hemodynamic information provided by different modalities. For hemoglobin-related biomarkers provided by optical or MRI (BOLD) techniques, including *HbR*, *HbO*_2_, *HbT*, *sO*_2_, heart rate, or pulsatility, the inter-modality relationship may be considered either direct or indirect, depending on whether equivalent physiological parameters are assessed under comparable measurement conditions. Even when similar biomarkers are measured, differences in spatial resolution and imaging depth can limit direct one-to-one correlation, resulting in an indirect relationship. Vascular morphology, density, and perfusion-related biomarkers provided by LSCI, US Doppler or ULM techniques are more appropriately regarded as complementary measures because they reflect distinct structural and hemodynamic characteristics of the vasculature.

## 3. Photoacoustic Vascular and Hemodynamic Imaging Studies with Reference Modalities

### 3.1. PAI Combined with NIRS

Yang et al. (2020) demonstrated PAI for monitoring forearm skeletal-muscle hemodynamics by comparison with NIRS during arterial and venous cuff occlusion in healthy volunteers [73]. PA measurements were acquired at 760, 800, and 840 nm, whereas NIRS measurements were acquired at 780 and 840 nm. PA-derived *sO*_2_ and NIRS-derived *sO*_2_ were estimated using least-squares spectral fitting and the modified Beer–Lambert law, respectively. During venous occlusion, PA amplitude and NIRS-derived *HbT* progressively increased with blood accumulation, while muscle *sO*_2_ values measured by PA and NIRS were comparable at 54.9 ± 1.12% and 58.2 ± 4.38%, respectively (Figure 3a). The temporal hemodynamic responses measured by PA were generally consistent with NIRS measurements. Rather than providing direct validation of absolute hemodynamic parameters, NIRS was used as a complementary modality to compare temporal changes in muscle blood volume and oxygenation during the cuff-occlusion protocol.

Hassan et al. (2023) demonstrated complementary PAI and continuous-wave NIRS for longitudinal monitoring of tissue responses at an implant site [74]. Spectral PA images were acquired at wavelengths ranging from 680 to 970 nm and processed using the SPAX framework with spectral decomposition, US-guided segmentation, and fluence correction to estimate *HbO*_2_, *HbR*, and *sO*_2_ (Figure 3b). CW-NIRS measurements at 740 and 840 nm were analyzed using the modified Beer–Lambert law to estimate relative changes in *HbO*_2_, *HbR*, and tissue oxygen saturation (*StO*_2_). During longitudinal monitoring, both modalities showed generally consistent changes in hemoglobin composition and tissue oxygenation associated with the implant response (Figure 3c). Rather than quantitatively validating PA-derived oxygenation, NIRS provided a complementary semiquantitative reference for temporal tissue changes. The study demonstrates the benefit of combining spatially resolved PAI with longitudinal NIRS-based oxygenation monitoring.

More recently, Qu et al. (2026) demonstrated PAI using a Nine-Grid Segmentation Strategy (NGSS) to characterize heterogeneous oxygenation responses in the thenar muscle during vascular occlusion [75]. PA images acquired at 700 and 1064 nm were used to estimate tissue oxygenation based on the differential absorption of *HbO*_2_ and *HbR*. NGSS classified nonarterial pixels according to their *sO*_2_ changes during occlusion and reperfusion, generating nine functional phenotypes of local oxygen consumption and recovery. PA-derived responses were compared with spatially averaged NIRS-derived *StO*_2_ and transcutaneous oxygen (*TcPO*_2_) measurements. While the reference modalities reflected overall temporal oxygenation changes, PA additionally revealed spatial heterogeneity in microcirculatory responses. Thus, NIRS and *TcPO*_2_ were used as complementary references for comparison with PA-derived *sO*_2_.

Beyond NIRS-based comparisons, Merdasa et al. (2021) evaluated hyperspectral PAI against diffuse reflectance spectroscopy (DRS) during finger ischemia and reperfusion in healthy volunteers [76]. Hyperspectral PA and ultrasound imaging over 680–970 nm were combined with US-guided segmentation of the epidermis, dermis, and hypodermis. Linear spectral unmixing of *HbO*_2_, *HbR*, melanin, fat, and water was used to generate pixel-wise *sO*_2_ and depth-resolved oxygenation profiles. DRS measurements were acquired continuously as a complementary reference during occlusion and reperfusion. Both modalities showed similar temporal oxygenation responses during ischemia and recovery. Rather than providing direct quantitative validation of PA-derived *sO*_2_, DRS was mainly used to assess agreement in temporal oxygenation changes.

Li et al. (2026) introduced MOBILE, a miniaturized broadband PAI system for continuous monitoring of microvascular responses during physiological activity [77]. Dual-wavelength PAI at 532 and 1064 nm was used to assess relative hemoglobin changes and tissue oxygenation, with the ratio of PA signals at 1064 and 532 nm defined as an oxygenation-sensitive S factor. During squatting and cycling, depth-resolved changes in the S factor were compared with *TcPO*_2_, while heart rate from a sports watch provided complementary information on systemic responses. PA-derived S factors and *TcPO*_2_ showed consistent temporal trends during exercise and recovery. Rather than providing direct quantitative validation of *sO*_2_, *TcPO*_2_ and heart-rate measurements served as complementary physiological references for interpreting relative oxygenation and systemic exercise responses.

Overall, NIRS and related optical monitoring techniques provide valuable physiological references for assessing tissue oxygenation and hemoglobin-related responses alongside PAI. These modalities typically offer high temporal sensitivity but limited spatial localization and shallow depth, whereas PAI enables spatially resolved and depth-dependent visualization of vascular and oxygenation changes. Across the reviewed studies, PA-derived responses generally exhibited temporal patterns consistent with those obtained using NIRS, DRS, or *TcPO*_2_ during vascular occlusion, exercise, and longitudinal monitoring. Nevertheless, direct assessment is constrained by differences in sampling depth, the localized image-based analysis employed in PAI, and variations in optical fluence. Furthermore, NIRS-derived *StO*_2_, DRS-based oxygenation estimates, and *TcPO*_2_ are physiologically related to, but not directly equivalent to, PA-derived *sO*_2_. These relationships are therefore more appropriately interpreted as indirect assessments that provide complementary physiological context. Future studies incorporating physiologically relevant reference measurements under controlled experimental conditions may further strengthen confidence in the reliability and physiological interpretation of PA-derived oxygenation biomarkers. All reviewed PAI studies complemented with NIRS are summarized in Table 1.

### 3.2. PAI Combined with PPG

Ahn et al. (2022) developed an integrated PAM–PPG system to monitor vascular motion and pulsatile blood-volume changes in healthy volunteers during vascular cuff occlusion (Figure 4a) [39]. PA and PPG signals were acquired alternately during the forward and reverse scans of the galvanometer using a 532 nm pulsed laser and light-emitting diode (LED), respectively. PAM tracked the axial position of a vessel to measure cardiac-induced vascular motion, while PPG monitored pulsatile blood-volume changes. Under normal conditions, PAM- and PPG-derived heart rates agreed at 1.35 Hz (81 BPM), with temporally aligned pulsatile signals, whereas both vessel motion and the PPG waveform were markedly suppressed during vascular occlusion (Figure 4b). These results demonstrated complementary monitoring of vascular motion and pulsatile hemodynamics using PAM and PPG. However, the limited scanning range and susceptibility of motion-based measurements to subject movement remained important limitations, while the use of a single wavelength precluded functional measurements such as *sO*_2_.

In a follow-up study, Ahn et al. (2023) applied high-speed PAM to an acute hyperglycemic rat model to evaluate transient arterial vasoconstriction in response to increased blood glucose over 150 min [78]. Repeated PAI of the rat ear was used to quantify changes in vessel diameter following glucose administration, while blood glucose levels were monitored using a glucometer, and PPG was used to monitor heart rate and respiratory rate. Representative PA maximum amplitude projection (MAP) and diameter-mapped images at the 7th and 30th measurements demonstrated temporal changes in the vasculature (Figure 4c). PAM measurements showed that arteriole diameter decreased by 5.7 ± 1.1% in the glucose group, whereas no significant change was observed in venules. Heart rate and respiratory rate remained stable throughout the experiment (Figure 4d). Rather than directly validating the PA-derived vascular measurements, the glucometer confirmed changes in blood glucose levels, while PPG served as an auxiliary modality for monitoring physiological stability. PAM provided direct visualization and quantification of the associated vascular changes.

Overall, PPG provides a useful physiological reference for assessing pulsatile vascular dynamics alongside PAI. PPG offers high temporal sensitivity to pulse-related changes but does not provide spatially resolved vascular information, whereas PAI enables localized imaging of vascular structure and motion. Vascular pulsatility and heart rate are suitable for direct assessment, as corresponding temporal information can be independently derived from both PA signals and PPG waveforms. In contrast, PA-derived *sO*_2_ and PPG-derived *SpO*_2_ represent physiologically related but non-equivalent oxygenation measurements and are therefore more appropriately interpreted as an indirect assessment. PA-derived vessel diameter and morphology provide additional complementary structural information that is not available from conventional PPG. In the integrated PAM–PPG study, differences between the measurements may arise from their distinct signal origins, alternating acquisition scheme, and sensitivity to motion, as PAM tracks vessel displacement while PPG detects pulsatile changes in blood volume. The limited scanning range and single-wavelength acquisition of the PAM system further restricted broader vascular assessment and functional measurements such as *sO*_2_. Future studies combining temporally synchronized PPG measurements with spatially resolved PA imaging may provide a more comprehensive assessment of vascular dynamics and improve the physiological interpretation of PA-derived vascular biomarkers. All reviewed PAI studies complemented with PPG are summarized in Table 2.

### 3.3. PAI Combined with LSCI and/or HSI

Qin et al. (2025) developed a cortex-wide multimodal microscope (multiScope) that integrates wide-field Ca^2+^ fluorescence microscopy, OR-PAM, and LSCI for simultaneous visualization of neuronal activity and hemodynamic responses in awake mice [79]. The system enables large-field-of-view imaging of the entire mouse cortex (8.6 mm diameter) with high spatial resolution and fast temporal sampling, allowing concurrent measurement of neural Ca^2+^ dynamics, *HbT*, and blood flow velocity. OR-PAM (559 nm) provided label-free, high-resolution visualization of cerebral vasculature and quantified *HbT* changes to represent blood volume dynamics, while LSCI provided wide-field cerebral blood flow velocity (CBFv) mapping based on speckle decorrelation. Using sensory stimulation and anesthesia challenge models, the authors investigated neurovascular coupling by correlating neuronal activation with hemodynamic responses. The study demonstrated that OR-PAM and LSCI provide complementary hemodynamic information, where PAI offers vessel-resolved *HbR* contrast whereas LSCI enables large-area perfusion monitoring. In a recent follow-up study, multiScope was used to evaluate the effects of anesthetics in seizure-related neurovascular responses [80].

Afzal et al. (2026) investigated age-dependent vascular recovery in a mouse model of peripheral artery disease (PAD) using multispectral optoacoustic tomography (MSOT), LSCI, and histological analysis (Figure 5a) [81]. PAD was induced by femoral artery cauterization in young (2-month-old) and aged (18-month-old) mice, followed by longitudinal imaging of the recovery process at baseline and 3, 7, and 14 days after ischemic injury. MSOT (680, 730, 760, 798, 874, 930, 972 nm) quantified *StO*_2_, while LSCI assessed relative blood perfusion, providing complementary measures of tissue oxygenation and blood flow. Both modalities demonstrated an acute reduction in vascular function after ischemia followed by progressive recovery, with substantially faster recovery in young mice than in aged mice. Histological analyses of collagen deposition, CD31-positive vascular structures, and Ki-67-positive proliferating cells corroborated the imaging findings and revealed enhanced angiogenesis and tissue regeneration in young animals. The study demonstrates that MSOT and LSCI provide complementary, noninvasive measures of *StO*_2_ and perfusion, respectively, and that their combination with histology can facilitate longitudinal assessment of age-dependent vascular responses in PAD.

Liu et al. (2020) developed an integrated PA–HSI dual-modal microscope for the characterization of melanoma and cutaneous squamous cell carcinoma (cSCC) injected into the mouse ear (Figure 5b) [82]. The hybrid platform combines OR-PAM (527 nm) and HSI, where both commonly provide vascular images but with different functional information of flow velocity and *sO*_2_, respectively. Notably, PAI was used to estimate the blood flow velocity from the PA Doppler bandwidth broadening effect instead of its conventional role of measuring *sO*_2_. Further, PA images were used to measure vascular morphological behaviors of the two cancers, quantified as microvascular density (MVD) and distance metric (DM). Finally, all metrics were combined into a single quantitative biomarker, metabolic rate of oxygen (*MRO*_2_), that appeared to diverge with significantly different trends in the two cancer models even in early growth stages (up to day 9).

Zhang et al. (2026) developed a dual-modal PA-HSI microscopy platform for real-time monitoring and adaptive control of vascular-targeted photodynamic therapy (V-PDT) [83]. HSI simultaneously acquired diffuse reflectance and fluorescence spectra to quantify *sO*_2_ and photosensitizer bleaching, while PAM (532 nm) provided detailed visualization of microvascular architecture for monitoring treatment-induced vascular damage. Using these complementary measurements, the authors established a dual-threshold irradiation strategy in which both *StO*_2_ and photosensitizer bleaching were monitored to determine when light delivery should be adjusted or terminated. To evaluate the therapeutic efficacy, various V-PDT protocols, including the dual-threshold approach, were compared in terms of vascular responses imaged via PAM. The study demonstrated that combining HSI-derived physiological feedback with PA-derived vascular information enabled individualized, physiology-guided control of photodynamic therapy, highlighting the complementary roles of HSI and PAM for longitudinal assessment of vascular function and treatment response.

Stridh et al. (2024) demonstrated a multimodal approach combining LSCI, HSI, and PAI to characterize a periorbital cutaneous angiosarcoma in an 82-year-old patient (Figure 5c) [40]. LSCI and HSI images were acquired in vivo before tumor excision, where LSCI provided a map of superficial blood perfusion while HSI provided information on tissue *HbT* and *sO*_2_. PAI (680–970 nm, 5 nm step) was subsequently performed ex vivo on the excised tumor (due to laser safety considerations) to obtain depth-resolved 3D maps of molecular composition, including *HbO*_2_, *HbR*, and collagen. The tumor exhibited markedly increased perfusion together with reduced *sO*_2_, suggesting pooling of deoxygenated blood within the highly vascularized angiosarcoma. PAI further provided depth-resolved visualization of the molecular composition and oxygenation of the tumor that could not be obtained from the superficial optical techniques alone. Rather than serving as a direct validation study, this proof-of-concept demonstrates the complementary nature of LSCI, HSI, and PAI, combining superficial perfusion and oxygenation measurements with depth-resolved molecular information.

Xie et al. (2025) developed a genetically engineered Janus polypeptide hydrogel designed to provide on-demand oxygenation and promote vascular regeneration for the treatment of infected diabetic wounds [84]. The therapeutic effects of the hydrogel were evaluated in vivo using a combination of HSI, PAI (532 nm), and LSCI, together with histological and molecular analyses. HSI was used to evaluate *HbO*_2_, *HbR*, and *sO*_2_, while PAI and LSCI were used to monitor changes in local blood perfusion or vascular status. The multimodal imaging results demonstrated enhanced perfusion and oxygenation in the hydrogel-treated wounds, consistent with improved angiogenesis and restoration of normal skin architecture. Together with histological evidence, these findings supported the ability of the engineered hydrogel to overcome the oxygen diffusion barrier and promote functional vascular regeneration.

In summary, LSCI and HSI provide optical measurements that can be compared with PA-derived vascular and hemodynamic information. HSI-derived *sO*_2_ can be directly compared with PA-derived *sO*_2_ when measurements are obtained from superficial tissue regions with comparable sampling conditions. However, the limited penetration depth and surface-sensitive characteristics of HSI and LSCI can restrict direct correspondence with depth-resolved PA measurements. LSCI-derived perfusion and other hemodynamic biomarkers are physiologically related to PA-derived vascular responses but represent different measurement quantities and are therefore more appropriately classified as indirect assessments. Differences in measurement principles, sampling depth, and the comparison of two-dimensional surface measurements with depth-resolved PA imaging may further contribute to inter-modality variability. Accordingly, HSI is particularly suitable for direct assessment of superficial oxygenation measurements, whereas LSCI and related optical biomarkers provide indirect or complementary information on vascular perfusion and hemodynamic changes. All reviewed PAI studies complemented with LSCI and HSI are summarized in Table 3.

### 3.4. PAI Combined with Doppler US

Giustetto et al. (2015) demonstrated a five-mode sequential imaging protocol combining US B-mode, color Doppler, power Doppler, pulsed-wave (PW) Doppler, and PAI for noninvasive visualization of deep cerebral vasculature in rats without skull resection [85]. US and Doppler imaging were performed using a 16 MHz linear-array transducer through the temporal and occipital cranial foramina, while multispectral PAI was acquired over 680–970 nm. B-mode US provided anatomical information on the brain parenchyma, color Doppler visualized blood-flow velocity and direction, power Doppler assessed vascular flow-related signals, and PW Doppler characterized vascular pulsatility and differentiated arteries from veins. PAI further provided hemoglobin-related vascular and oxygenation information. The combined approach enabled characterization of major cerebral vessels, including the circle of Willis, internal carotid arteries, and middle cerebral arteries. The Doppler modes provided complementary information on vascular flow dynamics, while PAI characterized hemoglobin-related vascular contrast and oxygenation. Image quality remained dependent on the cranial acoustic window and imaging plane.

Heres et al. (2017) evaluated a hand-held PA probe for assessing blood volume and superficial vascularization using power Doppler US (PDUS) and high-frequency B-mode US as reference modalities [86]. PAI was performed at 805 nm using a 7.5 MHz linear-array transducer. In tissue-mimicking perfusion phantoms, the PA signal area increased with increasing perfused cross-sectional area from 1.3% to 5.4%, showing a similar trend to PDUS and supporting the sensitivity of PAI to changes in blood volume. In human imaging, 3D PA and US imaging were further performed to visualize superficial vasculature. The vascular structures visualized with PA and high-frequency US showed comparable vascular structures in terms of vessel number and size. PDUS provided a reference for perfusion-related changes, whereas US was used to compare vascular geometry. Together, PAI provided high vascular specificity for superficial vessels, while US offered complementary information on vascular structure and perfusion.

Keša et al. (2021) demonstrated using longitudinal US and PAI to monitor tumor oxygenation and vascularization in three patient-derived xenograft (PDX) models of mantle cell lymphoma [87]. Imaging was performed using a Vevo 3100/LAZR-X system (FUJIFILM, VisualSonics, Inc., Canada) with a linear-array transducer operated at 30 MHz for PAI at 750 and 850 nm and at 24 MHz for color Doppler imaging. B-mode US was used to assess tumor morphology and volume; PAI quantified *StO*_2_; and color Doppler measured tumor vascularization based on microvascular area (MVA). PA-derived *sO*_2_ within viable tumor regions remained relatively stable during tumor growth, whereas total tumor *sO*_2_ and Doppler-derived MVA decreased with increasing tumor volume, indicating progressive hypoxia and reduced vascularization. Doppler-derived MVA was further compared with CD31 immunohistochemistry to assess vascular density. Differences between Doppler and histological measurements were attributed to the limited sensitivity of Doppler to small and low-flow vessels and differences in the tissue regions evaluated. Overall, PAI provided functional information on tumor oxygenation, while Doppler US provided complementary assessment of tumor vascularization.

Godefroy et al. (2023) demonstrated interleaved 3D multispectral photoacoustic fluctuation imaging (MS-PAFI) and ultrafast Doppler US for complementary assessment of vascular morphology, blood flow, and oxygenation [41]. Imaging was performed using an 8 MHz spherical matrix-array transducer, with multispectral PA acquisition over 680–900 nm yielding a FOV of approximately 8×8×8 mm^3^ (Figure 6a). PAFI enhanced visualization of vascular morphology by extracting blood-induced PA fluctuations, while MS-PAFI enabled quantitative estimation of vascular *sO*_2_. Power Doppler provided vascular morphology, whereas directional power Doppler and color Doppler characterized blood-flow direction and velocity, respectively. In vitro experiments demonstrated that MS-PAFI provided *sO*_2_ measurements consistent with conventional multispectral PAI while reducing limited-view artifacts. In chicken embryos, co-registered MS-PAFI and Doppler imaging further demonstrated differences in oxygenation between vessels with opposite flow directions (Figure 6b). MS-PAFI provided oxygenation information, while Doppler US characterized blood-flow dynamics. However, long acquisition times and fluence dependence remained important limitations for quantitative *sO*_2_ imaging.

Kamali et al. (2023) demonstrated multimodal PAI, Doppler US, and MRI to characterize hyperacute cerebral hemodynamic and tissue responses in mouse models of traumatic brain injury (TBI) and subarachnoid hemorrhage (SAH) [88]. PAI and Doppler imaging were performed using a 21 MHz linear-array transducer, with PA-derived *sO*_2_ estimated over 690–970 nm. PAI quantified cerebral oxygenation, while 3D color Doppler measured velocity-weighted volume of blood flow (VVF). At 15 min after injury, TBI resulted in 19.2% and 41.0% reductions in ipsilateral *sO*_2_ and VVF, respectively, with partial recovery of oxygenation by 3 h. In contrast, SAH produced larger reductions in *sO*_2_ and VVF of 43.9% and 85.0%, respectively, without significant oxygenation recovery. These hemodynamic differences were further supported by MRI, which showed predominantly focal hemorrhage, edema, and perfusion abnormalities after TBI, whereas SAH produced more diffuse tissue alterations and bilateral reductions in cerebral blood flow. Together, PA and Doppler US enabled longitudinal assessment of cerebral oxygenation and blood-flow changes, while MRI provided complementary characterization of tissue injury and cerebral perfusion.

Peng et al. (2023) demonstrated longitudinal PAI and Doppler US imaging for monitoring inflammatory progression in a rat model of adjuvant-induced arthritis (AIA) [89]. Imaging was performed using an automated 3D PA–Doppler US system with PA excitation at 750 nm. B-mode US provided anatomical information of the ankle joint, PAI assessed synovial hyperemia based on increased blood volume, and Doppler US characterized inflammation-related increases in blood flow. Both modalities detected progressive vascular changes during arthritis development; however, PAI showed an earlier and more statistically significant increase, with PA signal changes detectable before those observed with Doppler US. Histopathology further confirmed synovial inflammation at the affected joints. These findings indicate that PAI was more sensitive to early hyperemia, while Doppler US provided complementary assessment of increased blood flow during inflammatory progression.

In summary, Doppler US provides quantitative assessment of blood-flow dynamics, including flow velocity, direction, and pulsatility, whereas PAI provides spatially resolved information on vascular structure and hemoglobin-related oxygenation. These measurements reflect distinct physiological properties; therefore, Doppler-derived flow parameters and PA-derived oxygenation biomarkers are more appropriately interpreted as complementary rather than comparative measurements. PAI may provide incomplete visualization of vascular structures due to limited-view effects and optical attenuation, while Doppler US may exhibit reduced sensitivity to microvascular blood flow, especially for UFD. PA and Doppler measurements can also be integrated within the same US platform, enabling spatially co-registered assessment of vascular structure, blood flow, and oxygenation. This multimodal approach provides a more comprehensive characterization of vascular and hemodynamic status. All reviewed PAI studies complemented with Doppler US are summarized in Table 4.

### 3.5. PAI Combined with ULM

Zhao et al. (2023) introduced a hybrid PA and fast ULM approach for high-resolution assessment of renal vascular structure, blood flow, and tissue oxygenation [90]. Multispectral PAI at 750 and 850 nm was used to assess hemoglobin oxygenation, whereas ultrafast US data acquired at 500 Hz were processed for super-resolution localization of circulating microbubbles. A sparsity-based localization algorithm substantially reduced the number of frames required for ULM reconstruction, enabling rapid visualization of the renal microvasculature. ULM provided vessel morphology together with microvascular flow direction and velocity, while PAI provided complementary functional information on blood oxygenation. During an oxygen challenge, changes in renal blood-flow velocity showed a positive relationship with changes in PA-derived hemoglobin oxygenation. These results demonstrated the coupling between renal perfusion and oxygenation that could not be fully characterized using either modality alone. The study highlights the complementary strengths of PA and fast ULM for assessing microvascular structure, flow dynamics, and functional oxygenation.

Building upon this PA/ULM framework, Zhao et al. (2025) developed label-free dual-modal photoacoustic and ultrasound localization imaging for longitudinal assessment of renal microcirculation during acute kidney injury [91]. Unlike conventional microbubble-based ULM, ultrafast US data were acquired at 500 Hz for 5 s at each imaging position and used to localize endogenous erythrocyte-derived signals, enabling super-resolution vascular imaging without exogenous contrast agents. PAI at 750 and 850 nm provided complementary measurements of hemoglobin composition and tissue oxygenation. The 3D label-free PA/UL imaging approach visualized renal microvascular structure, oxygenation, and blood-flow direction before and 72 h after ischemia–reperfusion surgery (Figure 7a). Renal vessel density, oxygenation, and relative blood volume were longitudinally evaluated before surgery and at 12, 24, 48, and 72 h after renal ischemia–reperfusion in both surgery and sham groups (Figure 7b). The surgery group showed progressive reductions in these parameters compared with the sham group, reflecting the development of renal microvascular dysfunction during acute kidney injury. The study demonstrates the progression of PA/ULM imaging from microbubble-based localization toward label-free assessment of vascular structure, blood volume, and oxygenation for longitudinal monitoring of renal disease.

Chen et al. (2024) developed a combined ultrafast ultrasound and PAI approach for multiparametric assessment of cerebral hemodynamics in rats [42]. Multispectral PA measurements at 750, 800, and 850 nm were used to estimate cerebral *sO*_2_, while ultrafast US provided Doppler-based measurements of cerebral blood volume (CBV) and CBF. The interleaved functional US and PA sequence operated at approximately 3.3 frames/s, enabling dynamic monitoring of CBV, CBF, and *sO*_2_ during hypercapnia stimulation. Microbubble-enhanced ULM was additionally performed using compounded US images acquired at approximately 700 Hz to generate super-resolved cerebral vascular maps and quantify blood-flow direction and velocity (Figure 7c). During hypercapnia, coordinated changes in relative cerebral blood volume (rCBV) and *sO*_2_ were observed within selected cortical regions, revealing distinct local cerebrovascular responses (Figure 7d). The study demonstrates that combined ultrafast US, ULM, and PAI can provide complementary information on vascular structure, perfusion, blood flow, and oxygenation for multiparametric assessment of cerebral hemodynamics.

Tang et al. (2024) developed three-dimensional integrated PA tomography and ULM noninvasive deep-brain hemodynamic imaging [92]. Multispectral PAI at 700, 750, and 1064 nm was used to assess cerebral oxygenation, whereas 3D ultrafast US data were acquired at approximately 215 volumetric frames/s for ULM reconstruction. Localization of circulating microbubbles generated super-resolved three-dimensional vascular maps and enabled measurements of vascular density and blood-flow velocity. PAI provided complementary information on hemoglobin-dependent optical contrast and *sO*_2_ throughout the cerebral vasculature. In a mouse ischemic stroke model, the affected hemisphere exhibited reductions in vascular density, blood-flow velocity, and *sO*_2_ compared with the contralateral hemisphere. These multimodal measurements enabled structural and functional consequences of cerebral ischemia to be evaluated within the same vascular network. The study demonstrates that 3D-PAULM can integrate vascular morphology, perfusion, blood-flow dynamics, and oxygenation for comprehensive assessment of deep-brain hemodynamics.

In summary, ULM provides super-resolved information on microvascular morphology and blood flow, whereas PAI provides spatially resolved vascular and oxygenation information based on optical absorption. Vascular morphology and density can be compared across the two modalities; however, ULM provides complementary high-resolution microvascular information through microbubble localization and tracking. Flow velocity can also be directly compared when corresponding vessels are assessed, although PA-based velocity estimation may be constrained by the laser pulse repetition frequency (typically in tens of Hz for PAT), which can limit the measurable velocity range. Conventional ULM requires microbubble administration and sufficient accumulation and tracking, which may prolong the acquisition time. Differences in contrast mechanisms and spatial resolution may also result in variations in vessel detectability and apparent vascular density between the modalities. The integration of ULM and PAI therefore enables complementary assessment of microvascular structure, blood flow, perfusion, and oxygenation. All reviewed PAI studies complemented with ULM are summarized in Table 5.

### 3.6. PAI Combined with MRI

Rich et al. (2015) evaluated the capability of multispectral PAI to quantify dynamic tumor oxygenation by validating against 4.7 Tesla BOLD MRI [93]. Mice were injected with human head and neck carcinoma cells and subjected to an oxygen breathing challenge using carbogen (95% O_2_, 5% CO_2_) while sequential PAI and BOLD MRI measurements were performed [93]. PAI was used to estimate *HbO*_2_, *HbR*, *HbT*, and *sO*_2_, whereas BOLD MRI monitored changes in *HbR*-induced magnetic susceptibility. Both modalities consistently detected increases in tumor oxygenation during hyperoxic breathing of carbogen followed by decreases back to baseline in room air inhalation, demonstrating comparable temporal hemodynamic responses despite their fundamentally different imaging contrasts. Rather than serving as identical measurements of hemoglobin concentration, PAI and BOLD MRI provided consistency in the relative changes in the biomarkers. The sensitivity of detecting the oxygenation variations, however, differed much in the two modalities (e.g., +25% in PAI and +7% in BOLD MRI during carbogen inhalation). As suggested by the authors, one of the reasons could be the mismatch in the image acquisition location and difficulties in the direct spatial co-registration of the two modalities. Further, even though PAI in principle aims to calculate the absolute measurements of *sO*_2_, both PAI and BOLD MRI were only used to observe relative variations in *sO*_2_ compared to baseline due to practical limitations.

Chen et al. (2022) introduced the first fully integrated hybrid magnetic resonance and optoacoustic tomography (MROT) platform capable of concurrent structural and functional imaging of the mouse brain (Figure 8a) [43]. The system combined a customized MR-compatible volumetric optoacoustic tomography (OAT) insert, an integrated radiofrequency coil, and a dedicated image reconstruction and co-registration pipeline, enabling simultaneous acquisition of multispectral optoacoustic data and multiple MRI sequences, including T1-weighted imaging, magnetic resonance angiography (MRA), and BOLD MRI. Using an oxygen breathing challenge in healthy mice, the authors demonstrated real-time measurements of *HbO*_2_, *HbR*, *HbT*, and *sO*_2_ with OAT while simultaneously recording BOLD responses with MRI. The study emphasized the complementary strengths of the two modalities: MRI provided excellent anatomical definition and established functional reference through BOLD imaging, whereas OAT offered direct assessment of multiple hemodynamic biomarkers with substantially higher temporal resolution. Notably, the integrated MROT platform enabled synchronized acquisition of complementary physiological information and facilitated accurate spatial co-registration between vascular anatomy, tissue oxygenation, and functional MRI signals. This work represents a significant technological milestone toward multimodal neuroimaging and establishes a powerful framework for cross-validation of PAI-derived hemodynamic biomarkers using concurrent MRI measurements.

Ni et al. (2018) demonstrated the feasibility of MSOT for noninvasive monitoring of acute cerebral ischemia by simultaneously assessing tissue oxygenation and matrix metalloproteinase (MMP) activity in a transient middle cerebral artery occlusion (tMCAO) mouse model [94]. MSOT was used to quantify *HbO*_2_, *HbR*, and *sO*_2_ during arterial occlusion and after reperfusion, while an activatable molecular probe enabled imaging of MMP activity. Structural MRI was employed to co-register the MSOT data with anatomical brain structures and accurately localize ischemic lesions. During the occlusion period, MSOT detected a significant reduction in *StO*_2_ in the ischemic hemisphere, followed by partial recovery after reperfusion. At 48 h after reperfusion, elevated MMP activity was observed in the infarct region and was further confirmed by ex vivo NIR fluorescence imaging. Rather than directly validating quantitative hemodynamic parameters against MRI-derived biomarkers, this work used MRI as an anatomical reference to improve localization and interpretation of optoacoustic measurements. The study highlights the complementary strengths of MRI and MSOT, demonstrating that multimodal imaging can simultaneously characterize structural injury, functional hemodynamics, and molecular processes during the evolution of ischemic stroke.

To further improve the PAI-MRI co-registration, Zhang et al. (2023) developed a co-registered multimodal imaging workflow that combines PAT and MRI to obtain complementary anatomical, functional, and molecular information in vivo (Figure 8b) [95]. The authors designed a dedicated animal holder compatible with both PAT and MRI, and an image registration strategy that enabled sequential PAT/MRI acquisitions while preserving subject positioning for accurate spatial alignment. The approach was demonstrated in 4T1 tumor-bearing mouse models using multispectral PAT to visualize vascular anatomy and hemoglobin- or ICG-based contrast, and MRI sequences to provide high-resolution anatomical information and soft-tissue contrast. The co-registration pipeline successfully fused the datasets from both modalities, allowing vascular structures detected by PAT to be interpreted within the detailed anatomical framework of MRI.

In summary, BOLD MRI provides an oxygenation-sensitive signal that can be related to PA-derived oxygenation responses, although it does not directly measure *sO*_2_. Relative oxygenation changes observed during physiological or pathological challenges are therefore more appropriately interpreted as indirect assessments with PA-derived *sO*_2_. Structural MRI, including T2-weighted imaging, provides complementary anatomical information for localizing vascular and hemodynamic alterations identified by PAI. Differences in contrast mechanisms, spatial and temporal resolution, and spatial correspondence between sequential acquisitions may contribute to variability between the modalities. Concurrent MRI–PAI implementation may reduce temporal and spatial mismatch but requires additional hardware integration, including magnetic-field compatibility, optical and acoustic access, and synchronization between the two imaging systems. All reviewed PAI studies complemented with MRI are summarized in Table 6.

**Figure 8:**
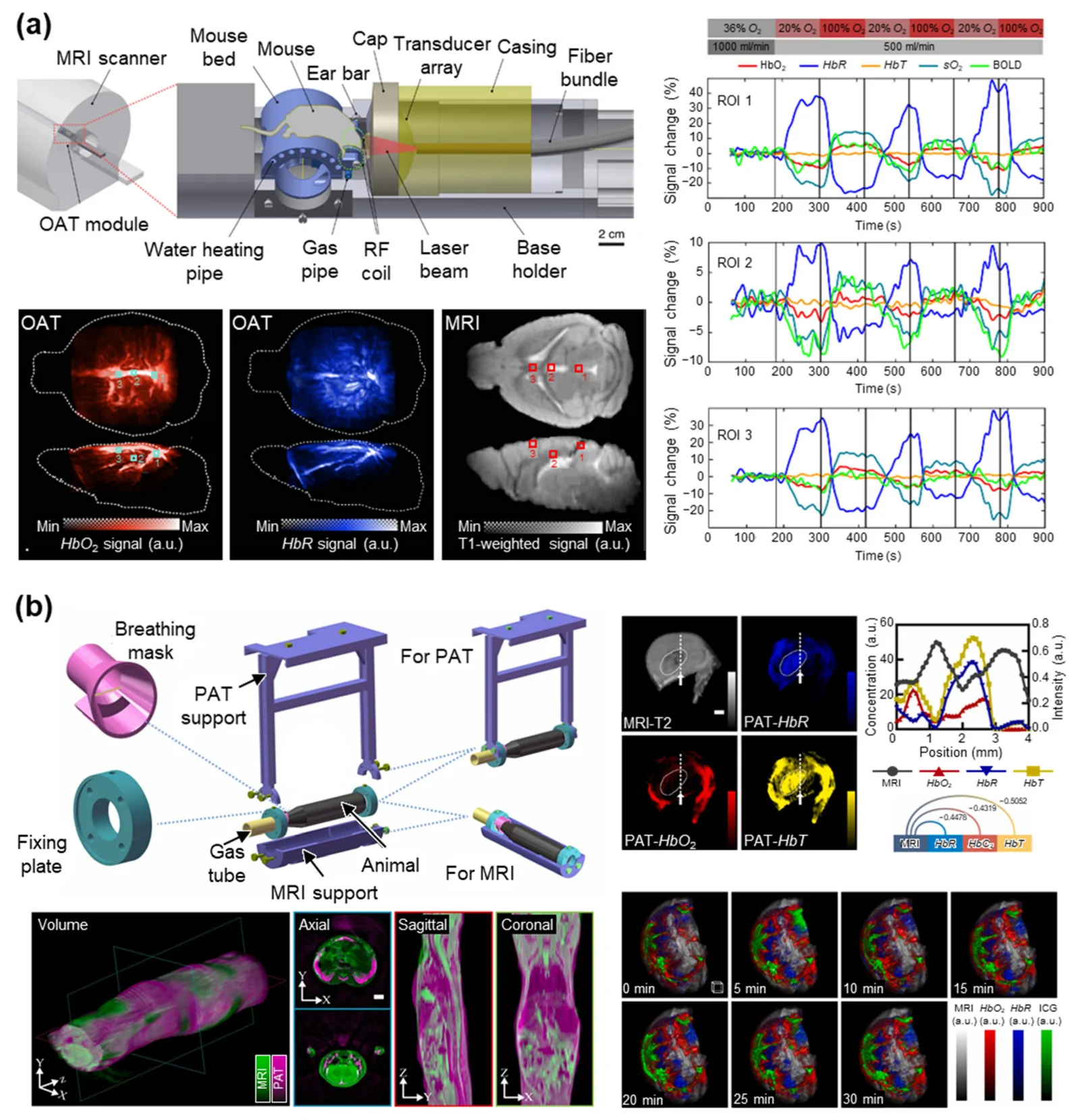
PAI research with MRI as reference modalities. (**a**) Fully integrated hybrid magnetic resonance and optoacoustic tomography (MROT) platform. A mouse brain was concurrently imaged with OAT and MRI during sequential breathing gas challenge. Temporal signal changes measured concurrently by OAT and MRI in regions of interest (ROIs) 1, 2, and 3 (square boxes) showed high concordance. (**b**) A dedicated animal holder compatible with both PAT and MRI, enabling precise image registration for sequential PAT/MRI. Images demonstrate co-registered PAT/MRI imaging of the whole mouse body, including cross-modal correlation of line profiles (dotted lines with arrows), and dynamic monitoring of hemoglobin and ICG in a 4T1 tumor-bearing mouse. MRI, magnetic resonance imaging; OAT, optoacoustic tomography; PAT, photoacoustic tomography; *HbO*_2_, oxyhemoglobin; *HbR*, deoxyhemoglobin; *HbT*, total hemoglobin; *sO*_2_, oxygen saturation; BOLD, blood-oxygen-level-dependent; ICG, indocyanine green; a.u., arbitrary unit. The images are reproduced with permission from refs. [43,95].

## 4. Current Challenges and Future Perspectives

Despite substantial advances in PA vascular and hemodynamic imaging, several challenges remain before the technology can achieve the level of maturity and clinical acceptance of established vascular imaging modalities. One of the fundamental challenges is the lack of a universal reference or gold standard for validating PA-derived vascular and hemodynamic measurements in vivo. While histology, ex vivo microscopy, and flow phantoms can provide valuable structural or functional references, they do not fully capture the dynamic and depth-dependent nature of vascular physiology in living subjects. This limitation is particularly relevant for human studies, where direct validation of small-vessel visibility, hemoglobin concentration, *sO*_2_, and perfusion is inherently difficult. Comparisons with established modalities such as MRI, Doppler ultrasound, and laser-based optical techniques can therefore provide important physiological references; however, these modalities measure different physical quantities and are themselves subject to modality-specific limitations. Future studies should move beyond qualitative evaluation toward rigorous cross-modal assessment and, where appropriate, quantitative validation using well-defined physiological endpoints and standardized experimental protocols.

Optical modalities, including NIRS, PPG, HSI, and LSCI, can provide hemoglobin-related oxygenation or superficial perfusion information with high temporal or spatial sensitivity. However, high-resolution optical imaging is predominantly restricted to superficial tissues, while spatial resolution progressively deteriorates or becomes poorly defined at greater depths because of strong optical scattering. These characteristics limit their ability to validate PA-derived oxygenation measurements in deeper tissues. In contrast, Doppler US, UFD, and ULM can visualize vascular structure and blood-flow dynamics at greater depths, in some cases with spatial resolution comparable to or higher than that of PAI. Their contrast mechanisms are primarily related to blood motion or microbubble localization and therefore provide few directly comparable metrics for validating PA-derived hemoglobin oxygenation. These techniques are particularly suitable for complementary assessment of vascular morphology and flow. MRI offers deep-tissue imaging and multiple structural and functional contrasts, with BOLD MRI providing oxygenation-sensitive information that can be compared with PA-derived *sO*_2_ changes. Nevertheless, BOLD contrast is not a direct measurement of *sO*_2_; its spatial resolution differs from that of PAI, and concurrent PA/MRI acquisition remains technically challenging. Apparent discrepancies between PA and reference measurements should therefore be interpreted in the context of differences in sampling volume, imaging depth, spatial and temporal resolution, and underlying contrast mechanisms. Concurrent hybrid systems and accurate image co-registration may improve cross-modal assessments by enabling different modalities to interrogate the same vascular structures and physiological states.

Based on our review of the literature, only a limited number of studies have developed PAI systems that are fully integrated with reference modalities, including the multiScope and MROT systems. The integration of multiple modalities remains technically challenging because each modality requires specialized hardware and acquisition schemes to maintain adequate signal quality while enabling co-registered data acquisition on a common imaging platform. Among the reference modalities, optical techniques such as NIRS, PPG, HSI, and LSCI are relatively amenable to integration with PAI through appropriate designs of sensor attachment and optical delivery around the PAI signal detection module, which typically requires direct contact with the target. Ultrasound techniques, including Doppler US and ULM, are particularly compatible with PAI because they can share the same imaging probe, facilitating their integration into commercial PA/US imaging platforms such as the VisualSonics Vevo LAZR system. In contrast, integration with MRI is considerably more challenging because of the requirements for MR-compatible hardware and the need to minimize potential cross-modal interference. Consequently, MRI and PAI are often acquired separately and subsequently co-registered, for example, using dedicated animal holders as demonstrated by Zhang et al. [95]. Although such approaches can provide spatial registration between the two modalities, they do not readily support real-time multimodal hemodynamic assessment. The MROT platform represents an important advance in this regard by enabling real-time, fully integrated MRI–PAI acquisition; however, further technological development is needed to facilitate its broader translation toward routine end-user applications.

In clinical applications, these challenges are further complicated by patient conditions, physiological motion, differences in sampling volume, and practical constraints related to system compatibility. Respiratory and cardiac motion, patient movement, probe pressure, and repositioning between sequential acquisitions may alter vascular geometry or physiological conditions, thereby reducing spatial and temporal correspondence between modalities. Furthermore, PAI commonly derives localized biomarkers from image-based regions of interest, whereas reference modalities may interrogate different or less spatially resolved tissue volumes, limiting direct numerical assessment even when the measured parameters are physiologically related. Concurrent acquisition may mitigate some of these uncertainties but requires appropriate synchronization, optical and acoustic access, hardware compatibility, and consideration of modality-specific safety and operational constraints. These factors should therefore be carefully considered when interpreting inter-modality relationships and designing future multimodal human studies.

These limitations highlight the importance of improving the intrinsic quantitative reliability of PAI in parallel with cross-modal assessment. PA-derived hemoglobin concentration and *sO*_2_ are affected by wavelength-dependent optical attenuation, heterogeneous fluence, spectral coloring, acoustic attenuation, and motion between wavelength acquisitions. In particular, spectral coloring can distort the measured PA spectrum with increasing imaging depth and lead to systematic errors in spectral unmixing and *sO*_2_ estimation [96,97,98]. Recent approaches have therefore used arterial oxygenation as an internal physiological reference to compensate for wavelength-dependent fluence variations and improve deep *StO*_2_ estimation [99]. Future studies should combine cross-modal assessment or quantitative validation, as appropriate, with rigorous correction of these physical and practical sources of error, including fluence compensation, motion correction, and system calibration aided by realistic simulation tools [100,101]. Given the absence of a universal reference modality, improving the intrinsic accuracy and robustness of PA measurements is essential for establishing reliable quantitative vascular and hemodynamic biomarkers.

Beyond endogenous hemoglobin-based PAI, exogenous contrast agents can further expand the applicability of PAI by enabling physiological and molecular imaging of living tissues, including the lymphatic system and tumors [10]. By exploiting their distinct optical spectral characteristics, these agents enable diverse imaging strategies that can overcome inherent physical limitations of PAI, such as deep-tissue imaging using NIR-II-absorbing agents, enhanced contrast with activatable agents, and super-resolution vascular imaging through agent localization. However, the clinical translation of contrast-enhanced PAI remains challenging because of the need for rigorous toxicological evaluation and regulatory approval of exogenous agents. One practical strategy is to repurpose clinically approved optical contrast agents, such as ICG and MB, or their derivatives, as PAI contrast agents, as demonstrated in several lymphatic imaging studies [20,102]. Importantly, the use of exogenous agents also introduces the need for reliable validation of their spectral separation and spatial distribution in PAI, particularly when multiple agents or complex tissue backgrounds are involved. In this regard, reference modalities with complementary contrast mechanisms can provide valuable independent validation of contrast-agent distribution and physiological responses. The combination of multifunctional contrast agents with multimodal PAI systems may therefore provide a promising framework for more reliable and comprehensive theranostic applications.

Finally, technical and methodological standardization remains an important barrier to reproducibility and clinical translation. PAI systems differ substantially in laser wavelength, pulse energy, pulse repetition rate, transducer configuration, acoustic bandwidth, acquisition sequence, beamforming, spectral unmixing, fluence correction, and quantitative analysis methods. Differences in physiological protocols, including oxygenation challenges, vascular occlusion, exercise, or pharmacological stimulation, further complicate comparisons across studies. Although initiatives such as the International Photoacoustic Standardization Consortium (IPASC) are working toward establishing common terminology, reporting practices, and measurement standards, broader adoption of standardized acquisition and analysis protocols is still needed [20,36]. Future research should establish consensus guidelines for reporting PA system specifications, exposure conditions, calibration procedures, quantitative biomarkers, physiological interventions, and validation metrics. Such standardization, combined with multicenter studies comparing PAI with complementary reference modalities and larger human studies, will be critical for determining the reproducibility, quantitative reliability, and clinical utility of PA vascular and hemodynamic biomarkers. Ultimately, the field should progress from demonstrating that PAI can visualize vascular and functional changes toward establishing how accurately, reproducibly, and clinically meaningfully these measurements reflect underlying human vascular physiology.

## 5. Conclusions

In conclusion, this review summarizes the use of PAI for vascular and hemodynamic assessment and its comparison with established reference modalities. PAI provides spatially resolved vascular and hemoglobin-related information, while optical techniques, Doppler US, ULM, and MRI provide complementary measurements of oxygenation, blood flow, perfusion, and vascular structure. However, differences in contrast mechanisms, imaging depth, and spatial and temporal resolution can limit direct quantitative assessment and complicate the selection of an appropriate reference modality. Improving quantitative reliability will therefore require appropriate cross-modal assessment, correction of physical and practical sources of error, and standardized acquisition and analysis protocols. Ultimately, larger clinical studies and, where appropriate, quantitative validation will be important for establishing PA-derived biomarkers as reliable tools for vascular assessment and longitudinal monitoring.

## Figures and Tables

**Figure 1:**
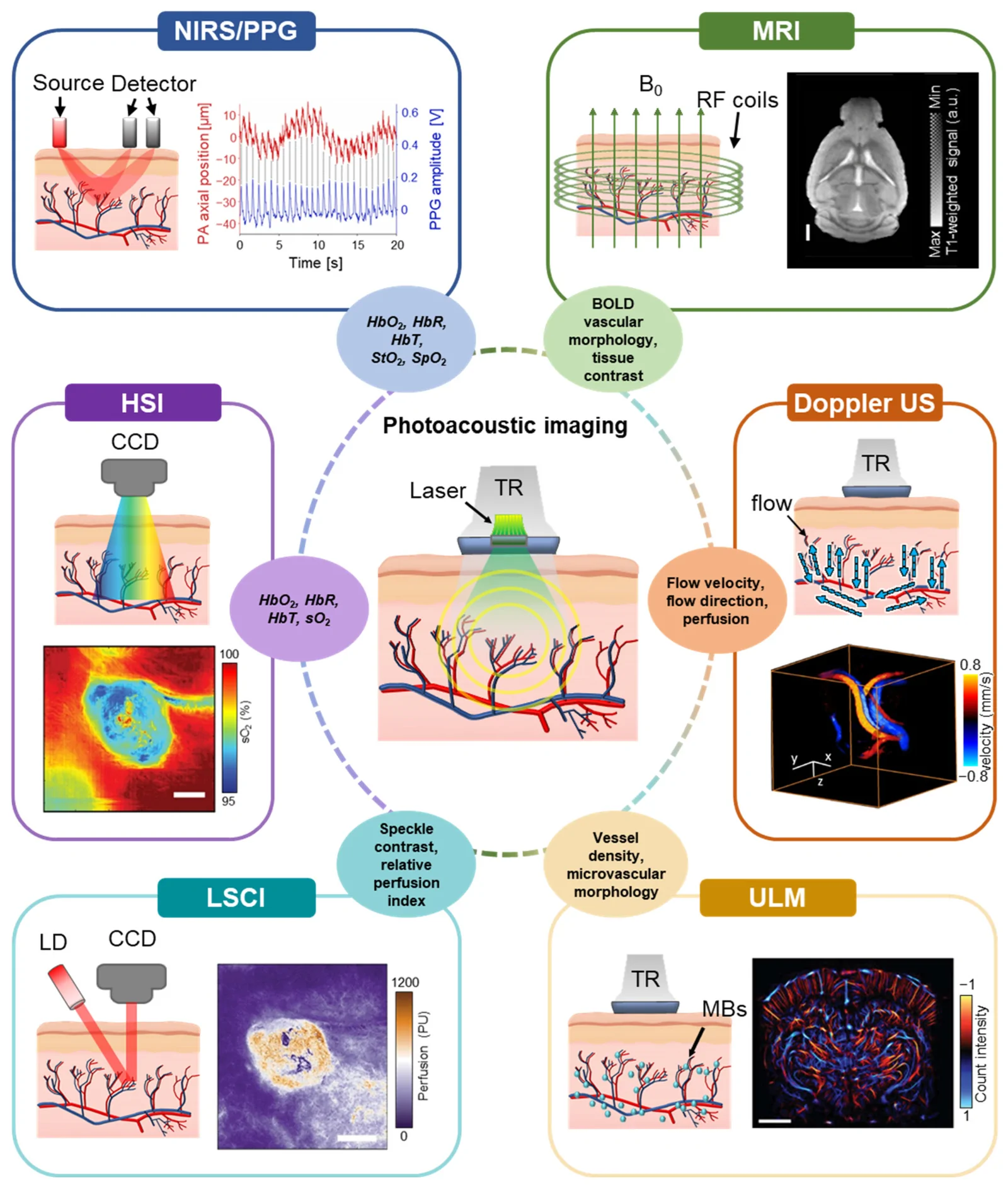
Complementary reference modalities for quantitative photoacoustic vascular and hemodynamic imaging. The NIRS/PPG, HSI, LSCI, Doppler US, ULM, and MRI images are reproduced from refs. [39,40,41,42,43] respectively. NIRS, near-infrared spectroscopy; PPG, photoplethysmography; MRI, magnetic resonance imaging; HSI, hyperspectral imaging; US, ultrasound; LSCI, laser speckle contrast imaging; ULM, ultrasound localization microscopy; *HbO*_2_, oxyhemoglobin; *HbR*, deoxyhemoglobin; *HbT*, total hemoglobin; *StO*_2_, tissue oxygen saturation; *SpO*_2_, peripheral oxygen saturation; *sO*_2_, oxygen saturation; LD, laser diode; CCD, charge-coupled device; BOLD, blood-oxygen-level-dependent; TR, transducer; RF, radio frequency; B_0_, static magnetic field; MBs, microbubbles.

**Figure 2:**
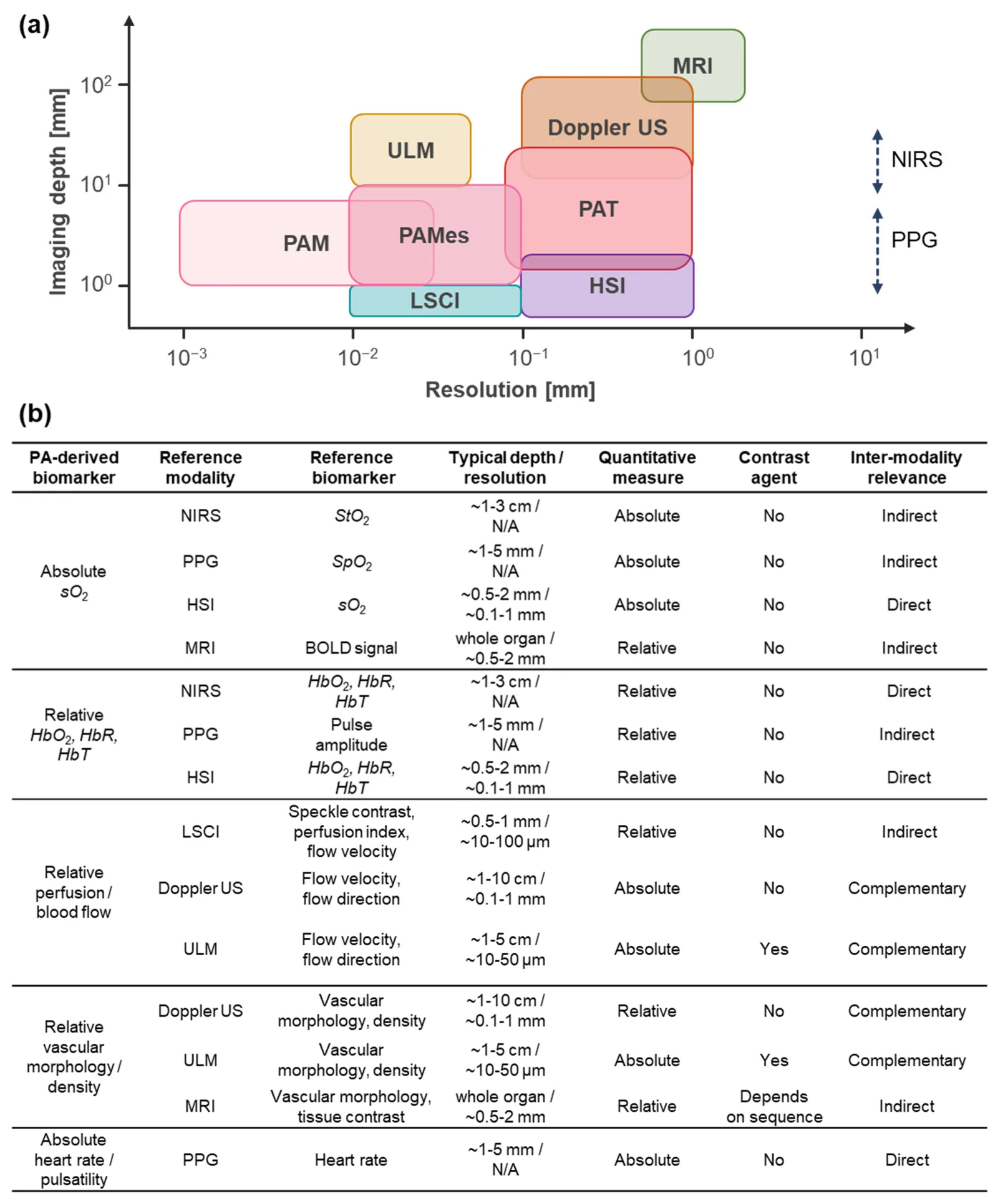
Basic characteristics and biomarkers of reference modalities. (**a**) Spatial resolution versus imaging depth of representative imaging modalities; NIRS and PPG are non-imaging optical sensing techniques without defined resolution and thus shown separately as arrows depicting the typical detectable depths. (**b**) Comparative table summarizing corresponding biomarkers, imaging or sensing depth, resolution, quantitative measure, and inter-modality relevance. NIRS, near-infrared spectroscopy; PPG, photoplethysmography; HSI, hyperspectral imaging; LSCI, laser speckle contrast imaging; US, ultrasound; ULM, ultrasound localization microscopy; MRI, magnetic resonance imaging; BOLD, blood-oxygen-level-dependent; PAM, photoacoustic microscopy; PAMes, photoacoustic mesoscopy; PAT, photoacoustic tomography; *HbO*_2_, oxyhemoglobin; *HbR*, deoxyhemoglobin; *HbT*, total hemoglobin; *sO*_2_, oxygen saturation; *SpO*_2_, peripheral oxygen saturation; N/A, not applicable.

**Figure 3:**
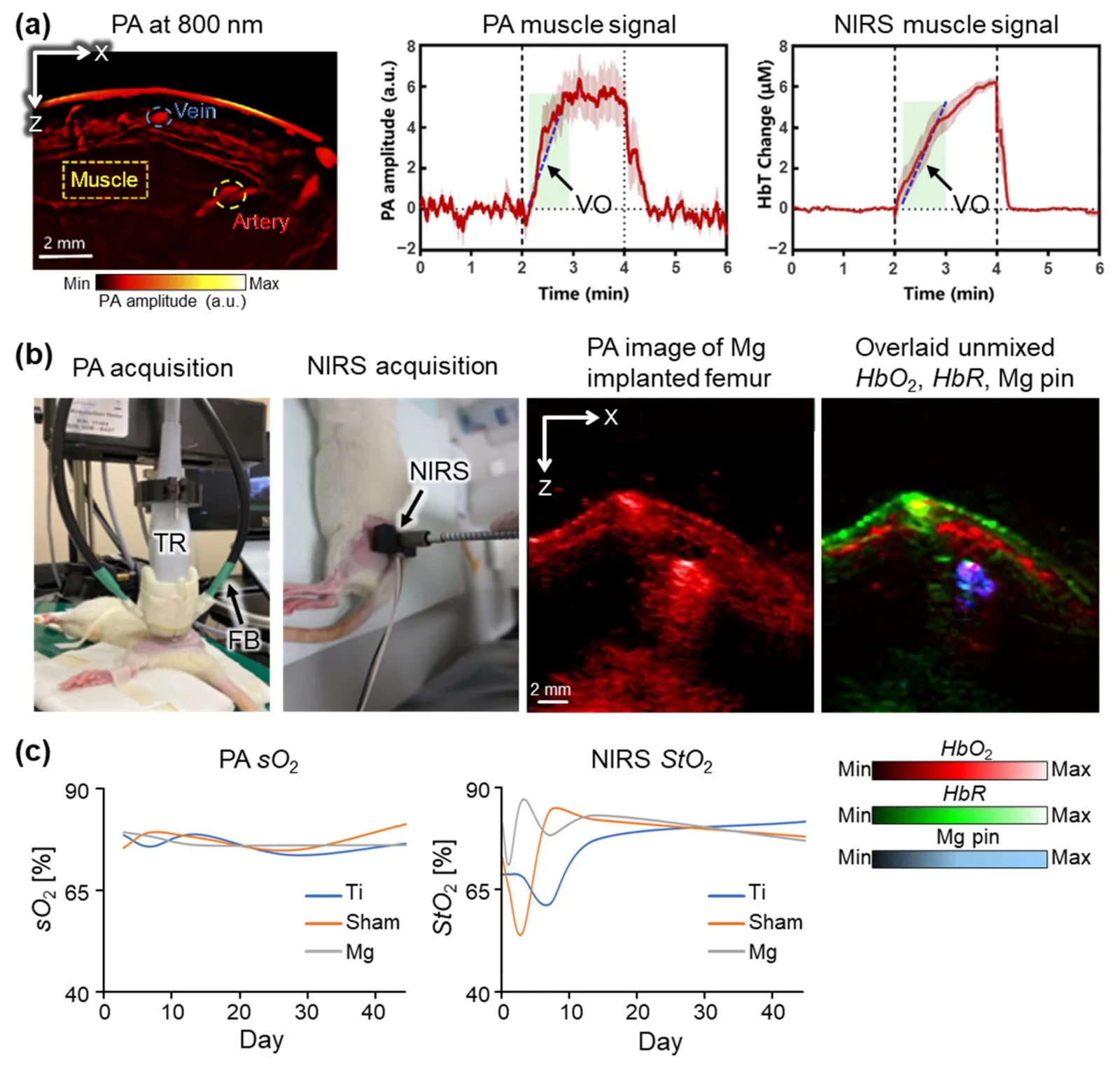
PAI research with NIRS as reference modalities. (**a**) PA image of the human forearm acquired at 800 nm and temporal PA and NIRS signals measured within the muscle ROI during venous occlusion. The blue dashed lines indicate the slopes of PA/NIRS signal changes during venous occlusion. (**b**) PA and NIRS measurement methodology in a rat femur implanted with Mg, including a representative PA image and an overlaid unmixed *HbO*_2_, *HbR*, and Mg-pin. (**c**) Comparative PA and NIRS analysis of oxygen saturation at the implant-tissue interface. VO, venous occlusion; TR, transducer; FB, fiber bundle; NIRS, near-infrared spectroscopy; *HbO*_2_, oxyhemoglobin; *HbR*, deoxyhemoglobin; Mg, magnesium; PA, photoacoustic; *sO*_2_, oxygen saturation; *StO*_2_, tissue oxygen saturation; Ti, titanium. The images are reproduced with permission from refs. [73] (Copyright (c) Optica Publishing Group) and [74].

**Figure 4:**
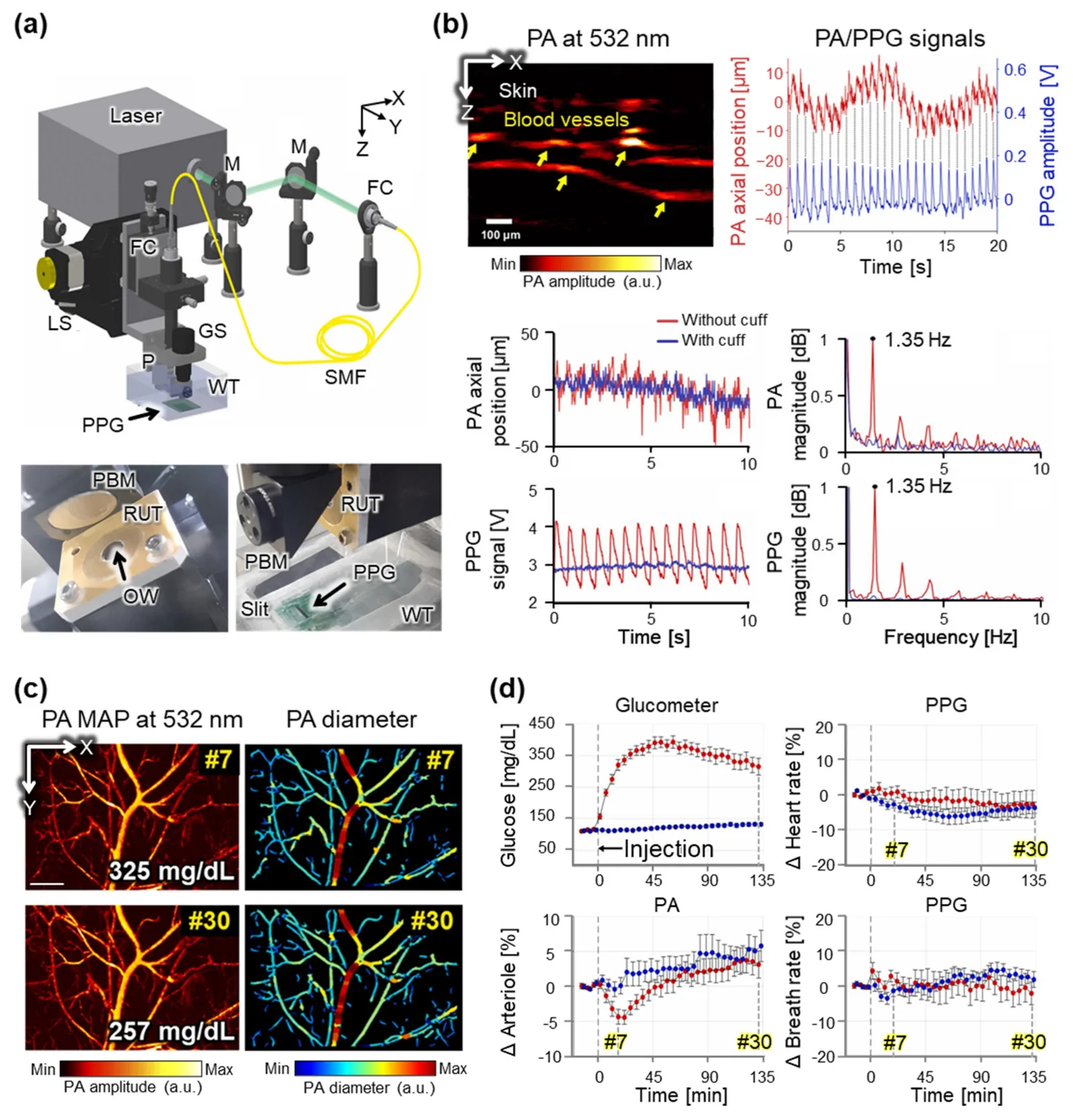
PAI research with PPG as reference modalities. (**a**) Schematic of the integrated PAM–PPG system combining an OR-PAM subsystem (532 nm pulsed laser, ring-shaped ultrasound transducer, parabolic-mirror-based scanning) with an LED-PD PPG module. (**b**) Cross-sectional PA B-mode image of a human finger showing skin and blood vessels, and quantification of vascular axial movement measured by PAM and PPG signal over time with and without cuff occlusion, along with their corresponding frequency spectra showing a dominant frequency of 1.35 Hz. (**c**) PA MAP and corresponding PA diameter-mapped vascular images of rat ear vasculature at the 7th and 30th measurements after glucose injection, with corresponding glucose levels. (**d**) Quantitative changes in glucose level (measured by glucometer), arteriole diameter (PA), and heart rate and breath rate (PPG) over time following glucose or PBS administration. The arrow indicates the injection site, and the yellow numbers indicate the measurement numbers corresponding to the images in (**c**). PAM, photoacoustic microscopy; PPG, photoplethysmography; OR-PAM, optical-resolution photoacoustic microscopy; LED, light-emitting diode; PD, photodiode; M, mirror; FC, fiber coupler/collimator; SMF, single-mode optical fiber; P, prism; GS, galvanometer scanner; WT, water tank; LS, linear stage; RUT, ring-shaped ultrasound transducer; OW, optical window; PBM, parabolic mirror; PA, photoacoustic; MAP, maximum amplitude projection; PBS, phosphate-buffered saline. The images are reproduced with permission from refs. [39,78].

**Figure 5:**
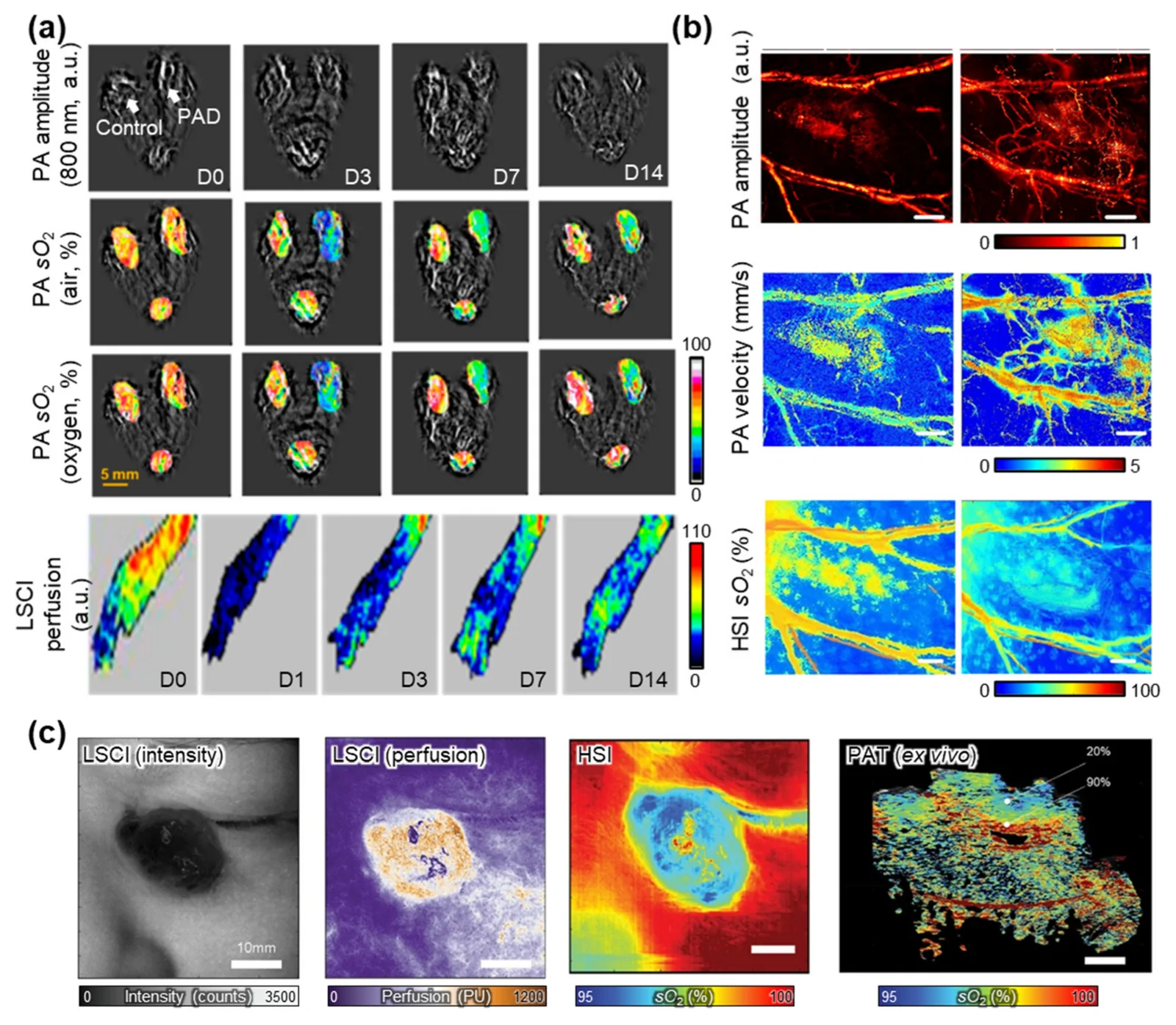
PAI research with LSCI and/or HSI as reference modalities. (**a**) PAI and LSCI images of hindlimbs of a mouse acquired during vascular recovery with different gas breathing challenges (air, oxygen) at days 0–14 after inducing peripheral artery disease. (**b**) PAI and HSI images of a mouse ear at days 3 and 9 after injection of skin cancer cells. (**c**) LSCI, HSI, and PAI images of a patient with periorbital cutaneous angiosarcoma. PA, photoacoustic; *sO*_2_, oxygen saturation; LSCI, laser speckle contrast imaging; HSI, hyperspectral imaging; a.u., arbitrary unit; PU, perfusion unit. The images are reproduced with permission from refs. [81,82] (Copyright (c) 2020 WILEY-VCH Verlag GmbH & Co. KGaA, Weinheim), and [40].

**Figure 6:**
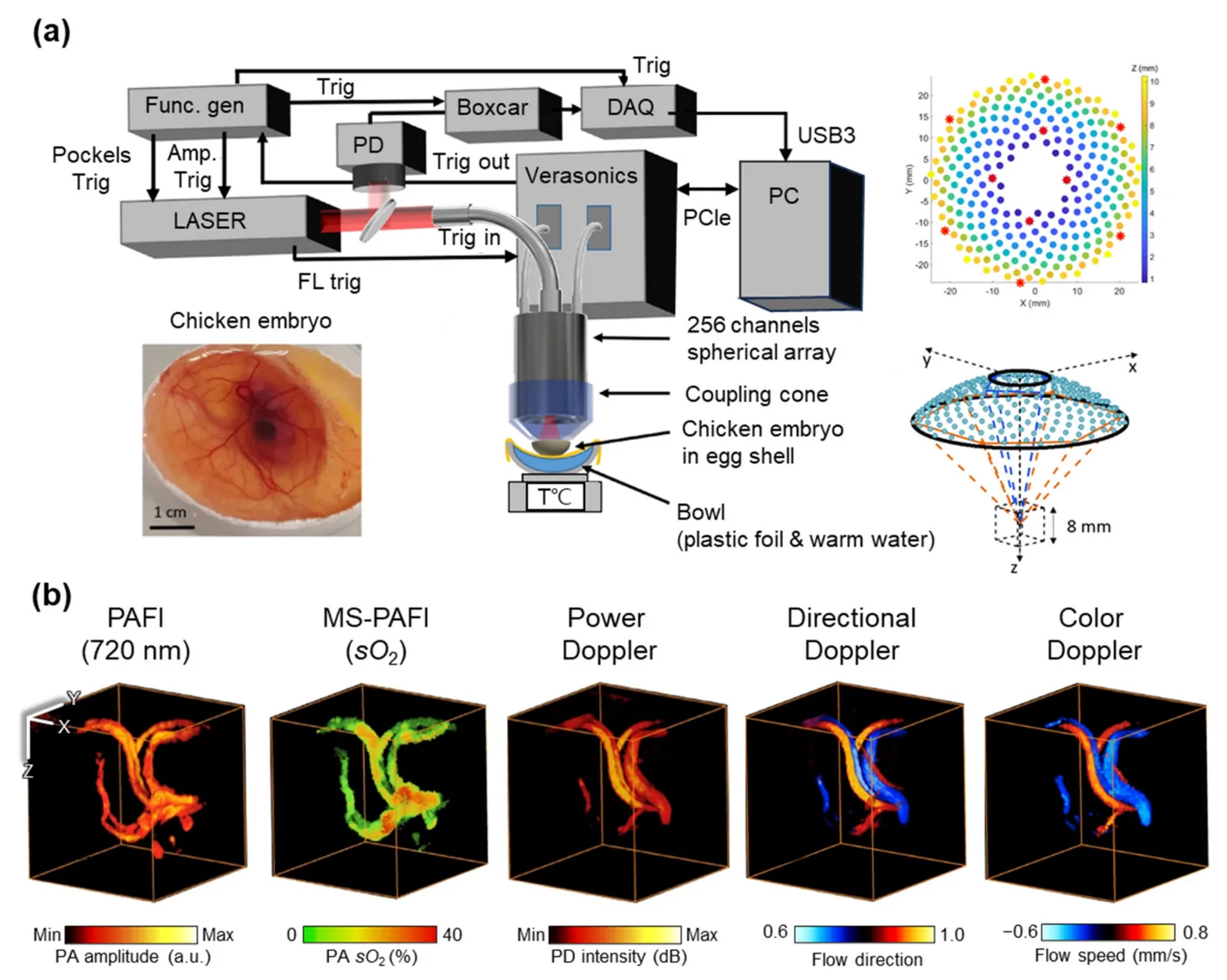
PAI research with Doppler US as reference modalities. (**a**) Three-dimensional images of blood vessels in chicken embryo obtained by PA, ultrasound Power Doppler, directional Power Doppler, and Color Doppler imaging. The red asterisks indicate the elements for US transmissions. (**b**) *sO*_2_ imaging using conventional DAS multispectral PA imaging and MS-PAFI; PA, photoacoustic; *sO*_2_, oxygen saturation; DAS, delay-and-sum; MS-PAFI, multispectral photoacoustic fluctuation imaging. The images are reproduced with permission from ref. [41].

**Figure 7:**
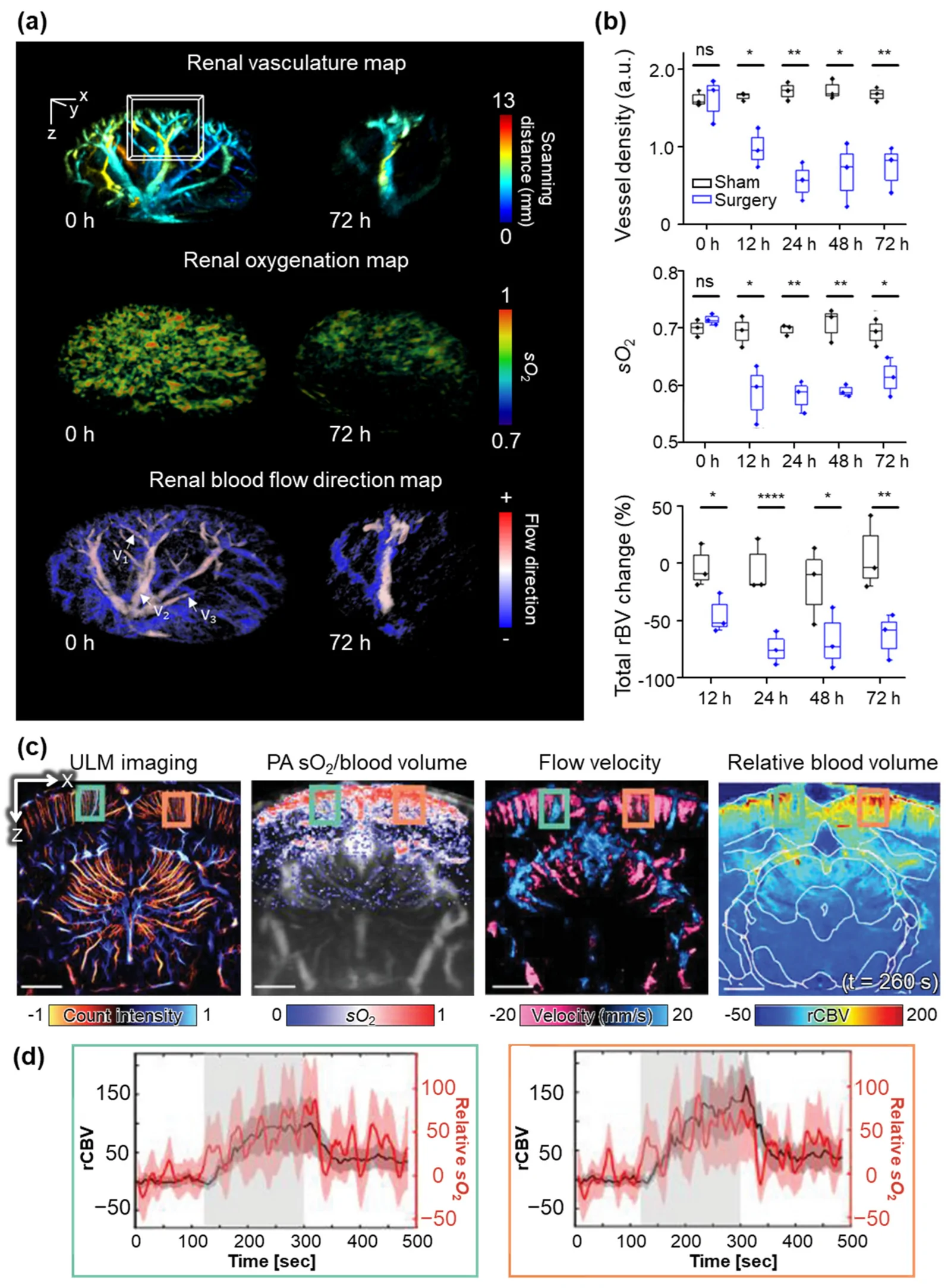
PAI research with ULM as reference modalities. (**a**) Label-free PA and ULM imaging of renal vasculature, oxygenation, and blood-flow direction before and 72 h after renal ischemia–reperfusion surgery in an acute kidney injury model. (**b**) Longitudinal comparison of renal vessel density, oxygenation, and total relative blood volume between the surgery and sham groups. (**c**) ULM-based super-resolved cerebral microvascular imaging, with an *sO*_2_ map obtained from multispectral PA imaging overlaid on the corresponding cerebral blood volume map, together with cerebral blood-flow and relative cerebral blood volume change maps during hypercapnia stimulation. The cyan and orange rectangles indicate the two cortical regions of interest. (**d**) Temporal changes in relative cerebral blood volume and relative *sO*_2_ in the two regions at selected time points during hypercapnia stimulation. *sO*_2_, oxygen saturation; rBV, relative blood volume; rCBV, relative cerebral blood volume; ULM, ultrasound localization microscopy; PA, photoacoustic, * *p* < 0.05, ** *p* < 0.01, **** *p* < 0.0001, ns, not significant. The images are reproduced with permission from refs. [42,91].

**Table 1:** Summary of PAI research with NIRS reference modalities. PAI, photoacoustic imaging; PAT, photoacoustic tomography; NIRS, near-infrared spectroscopy; DRS, diffuse reflectance spectroscopy; *HbO*_2_, oxyhemoglobin; *HbR*, deoxyhemoglobin; *HbT*, total hemoglobin; *sO*_2_, oxygen saturation; *StO*_2_, tissue oxygen saturation; *TcPO*_2_, transcutaneous oxygen; *pO*_2_, partial pressure of oxygen.

Ref.	PAI Modality	Reference Modality	Acquisition Scheme	Biomarkers		Imaging Target	Physiological Protocol
				PAI	Reference		
Yang et al. (2020) [73]	PAT (760, 800, 840 nm; custom-built)	NIRS (780, 840 nm; custom-built)	Separate system, sequential acquisition	*sO*_2_	*HbT*, *sO*_2_	Human forearm	Arterial and venous cuff occlusion, reperfusion
Hassan et al. (2023) [74]	PAT (680–970 nm, 5 nm step; Vevo LAZR-X)	NIRS (740, 840 nm; custom-built)	Separate system, sequential acquisition	*HbO*_2_, *HbR*, *sO*_2_	*StO*_2_	In vivo rat hindlimb/ implant-tissue interface	Longitudinal post-implantation monitoring
Qu et al. (2026) [75]	PAT (700, 1064 nm; Verasonics)	NIRS (760, 810, 840 nm; EnginMed N17), *TcPO*_2_ (Radiometer TCM4)	Separate system, sequential acquisition	*sO*_2_	*StO*_2_, *pO*_2_	Human thenar muscle	Vascular cuff occlusion, reperfusion
Merdasa et al. (2021) [76]	PAT (680–970 nm, 5 nm step; Vevo LAZR-X)	DRS (500–650 nm; moorVMS-oxy)	Separate system, sequential acquisition	*HbO*_2_, *HbR*, *sO*_2_	*sO*_2_	Human finger	Finger cuff occlusion, reperfusion
Li et al. (2026) [77]	PAT (532, 1064 nm; custom-built)	*TcPO*_2_, (Radiometer TCM4), Sport watch (Huawei GT3)	Separate system, concurrent acquisition	*HbR*, S factor vessel diameter	*pO*_2_, heart rate	Human finger, palm	Venous cuff occlusion, thermal challenge, exercise

**Table 2:** Summary of PAI research with PPG reference modalities. PAI, photoacoustic imaging; PAM, photoacoustic microscopy; PPG, photoplethysmography; PA, photoacoustic.

Ref.	PAI Modality	Reference Modality	Acquisition Scheme	Biomarkers		Imaging Target	Physiological Protocol
				PAI	Reference		
Ahn et al. (2022) [39]	PAM (532 nm; custom-built)	PPG (532 nm; custom-built)	Integrated system, concurrent acquisition	PA signal amplitude, vessel pulsation, heart rate	PPG signal amplitude, heart rate	Human finger	Vascular cuff occlusion
Ahn et al. (2023) [78]	PAM (532 nm; OptichoM)	PPG (532 nm; custom-built)	Integrated system, concurrent acquisition	Arteriolar and venular diameter change	Heart rate, breath rate	In vivo rat ear vasculature	Acute hyperglycemia induction

**Table 3:** Summary of PAI research with LSCI and/or HSI as reference modalities. PAI, photoacoustic imaging; OR-PAM, optical-resolution photoacoustic microscopy; PAT, photoacoustic tomography; LSCI, laser speckle contrast imaging; HSI, hyperspectral imaging; *HbO*_2_, oxyhemoglobin; *HbR*, deoxyhemoglobin; *HbT*, total hemoglobin; *sO*_2_, oxygen saturation.

Ref.	PAI Modality	Reference Modality	Acquisition Scheme	Biomarkers		Imaging Target	Physiological Protocol
				PAI	Reference		
Qin et al. (2025) [79]	OR-PAM (559 nm; custom-built)	LSCI, fluorescence microscopy (custom-built)	Integrated system, sequential acquisition	*HbT*, vascular morphology	Flow velocity, Ca^2+^ fluorescence	In vivo awake mouse whole-cortex	Sensory stimulation and anesthesia challenge to evaluate neurovascular coupling
Afzal et al. (2026) [81]	PAT (680, 730, 760, 798, 874, 930, 972 nm; In-Vision 256)	LSCI (PeriFlux 6000)	Separate system, sequential acquisition	*sO*_2_	Perfusion	In vivo mouse model for peripheral artery disease	Ischemia and recovery, gas breathing challenge (room air, oxygen)
Liu et al. (2020) [82]	OR-PAM (527 nm; custom-built)	HSI (custom-built)	Integrated system, sequential acquisition	Vascular morphology, flow velocity	*sO*_2_	In vivo mouse ear with melanoma and cutaneous squamous cell carcinoma	Tumor progression
Zhang et al. (2026) [83]	OR-PAM (532 nm; custom-built)	HSI (custom-built)	Separate system, sequential acquisition	Vascular morphology	*sO*_2_, photosensitizer bleaching	In vivo mouse chronic dorsal skinfold window chamber model	Vascular-targeted photodynamic therapy
Stridh et al. (2024) [40]	PAT (680–970 nm, 5 nm step; Vevo LAZR-X)	HIS (custom-built), LSCI (PeriCam PSI NR)	Separate system, sequential acquisition	*HbO*_2_, *HbR*, collagen	HSI: *HbT*, *sO*_2_ LSCI: perfusion	Cutaneous angiosarcoma patient (LSCI, HSI: in vivo; PAT: ex vivo)	-
Xie et al. (2025) [84]	Unknown (532 nm)	HSI, LSCI	Separate system, sequential acquisition	Angiogenesis, tissue perfusion	HSI: *HbO*_2_, *HbR*, *sO*_2_ LSCI: flow velocity	In vivo rat with *S.* *Aureus*-infected diabetic wound	Treatment recovery

**Table 4:** Summary of PAI research with Doppler US reference modalities. PAI, photoacoustic imaging; PAT, photoacoustic tomography; HFUS, high-frequency ultrasound; US, ultrasound; MRI, magnetic resonance imaging; *HbT*, total hemoglobin; *sO*_2_, oxygen saturation; MVA, microvascular area; VVF, velocity-weighted volume of blood flow; CBF, cerebral blood flow; PDX, patient-derived xenograft.

Ref.	PAI Modality	Reference Modality	Acquisition Scheme	Biomarkers		Imaging Target	Physiological Protocol
				PAI	Reference		
Giustetto et al. (2015) [85]	PAT (750, 850 nm; Vevo LAZR 2100)	HFUS, color/power/ pulsed wave Doppler (16 MHz; Vevo LAZR 2100)	Integrated system, sequential acquisition	Vessel morphology, *HbT*, *sO*_2_	Flow velocity, direction, pulsatility	In vivo rat brain	-
Heres et al. (2017) [86]	PAT (805 nm; Esaote MyLab One)	HFUS, Power Doppler, (7.5, 12 MHz; Esaote MyLab One)	Separated system, sequential acquisition	Perfused area, vessel geometry	Perfused area, vessel geometry	Perfusion phantom, in vivo human hand palm and wrist	-
Keša et al. (2021) [87]	PAT (750, 850 nm; Vevo 3100 LAZR-X)	HFUS, Color Doppler (24, 30 MHz; Vevo 3100 LAZR-X)	Integrated system, sequential acquisition	*sO*_2_	MVA, tumor volume	In vivo mouse subcutaneous PDX tumor	Longitudinal tumor growth monitoring
Godefroy et al. (2023) [41]	PAT (680–900 nm; Verasonics)	Power, directional, color Doppler, (8 MHz; Verasonics)	Integrated system, concurrent acquisition	Vessel morphology, *sO*_2_	Vessel morphology, flow direction, velocity	In vitro blood flow phantom, in vivo chicken embryo	-
Kamali et al. (2023) [88]	PAT (680–970 nm; Vevo 3100 LAZR-X)	HFUS, color Doppler, (21 MHz; Vevo 3100 LAZR-X), MRI (7T; Bruker)	Integrated system, sequential acquisition	*sO*_2_	VVF, hemorrhage, edema, diffusivity, CBF	In vivo mouse brain	Acute brain injury induction, longitudinal post-injury monitoring
Peng et al. (2023) [89]	PAT (750 nm; Vivid E95)	US, power Doppler (12.5 MHz; Vivid E95)	Integrated system, sequential acquisition	Total signal intensity, signal positive pixel count	Blood flow intensity, flow positive pixel count	In vivo rat ankle joint	Longitudinal arthritis progression monitoring

**Table 5:** Summary of PAI research with ULM reference modalities. PAI, photoacoustic imaging; ULM, ultrasound localization microscopy; PAT, photoacoustic tomography; *HbO*_2_, oxyhemoglobin; *HbR*, deoxyhemoglobin; *sO*_2_, oxygen saturation.

Ref.	PAI Modality	Reference Modality	Acquisition Scheme	Biomarkers		Imaging Target	Physiological Protocol
				PAI	Reference		
Zhao et al. (2023) [90]	PAT (750, 850 nm; Verasonics)	ULM (15 MHz; Verasonics)	Integrated system, concurrent acquisition	*HbO*_2_, *HbR*, *sO*_2_	Vessel structure, flow velocity and direction	In vivo mouse kidney/ popliteal lymph node	Oxygen challenge (20% O_2,_ 80% N_2_)
Zhao et al. (2025) [91]	PAT (750, 850 nm; Verasonics)	Label-free ULM (15 MHz; Verasonics)	Integrated system, concurrent acquisition	*HbO*_2_, *HbR*, *sO*_2_	Vessel density, blood volume, flow velocity	In vivo mouse kidney	Renal ischemia, reperfusion induced AKI
Chen et al. (2024) [42]	PAT (750, 800, 850 nm; Verasonics)	ULM/UFD (15 MHz; Verasonics)	Integrated system, concurrent acquisition	*sO*_2_	Blood perfusion, volume, flow velocity	In vivo rat brain	Hypercapnia (95% O_2_, 5% CO_2_)
Tang et al. (2024) [92]	PAT (700, 750, 1064 nm; Verasonics)	ULM (4 MHz; Verasonics)	Integrated system, sequential acquisition	*sO*_2_	Blood perfusion, vessel density, flow velocity	In vivo mouse whole brain	Permanent middle cerebral artery occlusion

**Table 6:** Summary of PAI research with MRI as reference modalities. PAI, photoacoustic imaging; MRI, magnetic resonance imaging; BOLD, blood-oxygen-level-dependent; TOF, time-of-flight; PAT (or OAT), photoacoustic (or optoacoustic) tomography; *HbO*_2_, oxyhemoglobin; *HbR*, deoxyhemoglobin; *HbT*, total hemoglobin; *sO*_2_, oxygen saturation; ICG, indocyanine green; MMP, matrix metalloproteinase.

Ref.	PAI Modality	Reference Modality	Acquisition Scheme	Biomarkers		Imaging Target	Physiological Protocol
				PAI	Reference		
Rich et al. (2015) [93]	PAT (750, 850 nm; Vevo LAZR)	BOLD MRI (4.7 T; Bruker Biospec with ParaVision 3.0.2)	Separate system, sequential acquisition	*HbO*_2_, *HbR*, *sO*_2_	BOLD signal	In vivo mice with head and neck tumors	Gas breathing response in tumors (95% O_2_, 5% CO_2_)
Chen et al. (2022) [43]	OAT (700, 730, 755, 800, 850 nm; custom-built)	BOLD MRI, TOF-MRA, T1 MRI (9.4 T; Bruker)	Integrated system, concurrent acquisition	*HbO*_2_, *HbR*, *HbT*, *sO*_2_	BOLD signal, vascular morphology	In vivo mouse brain	Gas breathing challenge (20–100% O_2_)
Ni et al. (2018) [94]	PAT (680, 685, 690, 695, 700, 715, 730, 760, 800, 850 nm; MSOT inVision128)	T2-weighted MRI (7 T; Bruker Pharmascan)	Separate system, sequential acquisition	*HbO*_2_, *HbR*, *sO*_2_, MMP signal	Infarct volume	In vivo mouse brain with transient middle cerebral artery occlusion	Occlusion and reperfusion
Zhang et al. (2023) [95]	PAT (700, 730, 760, 800, 850 nm; MSOT inVision128)	T1-/T2-weighted MRI (7 T; Bruker Pharmascan)	Separate system, sequential acquisition	*HbO*_2_, *HbR*, vascular morphology, ICG	Tissue contrast	In vivo healthy and tumor-bearing mice whole body	Perfusion monitoring after ICG injection

## Data Availability

No new data were created or analyzed in this study. Data sharing is not applicable to this article.

## Abbreviations

The following abbreviations are used in this manuscript:

PAI	Photoacoustic imaging
PA	Photoacoustic
*sO*_2_	Oxygen saturation
*HbO*_2_	Oxyhemoglobin
*HbR*	Deoxyhemoglobin
PAM	Photoacoustic microscopy
OR	Optical-resolution
AR	Acoustic-resolution
PAMes	Photoacoustic mesoscopy
PAT	Photoacoustic tomography
CT	Computed tomography
MRI	Magnetic resonance imaging
US	Ultrasound
PPG	Photoplethysmography
NIRS	Near-infrared spectroscopy
LSCI	Laser speckle contrast imaging
HSI	Hyperspectral imaging
*StO*_2_	Tissue oxygen saturation
ULM	Ultrasound localization microscopy
*SpO*_2_	Peripheral oxygen saturation
LD	Laser diode
CCD	Charge-coupled device
TR	Transducer
RF	Radio frequency
B_0_	Static magnetic field
MBs	Microbubbles
NIR	Near-infrared
*HbT*	Total hemoglobin
AC	Alternating current
DC	Direct current
UFD	Ultrafast Doppler
TOF	Time of flight
BOLD	Blood-oxygen-level-dependent
RBCs	Red blood cells
N/A	Not applicable
NGSS	Nine-Grid Segmentation Strategy
*TcPO*_2_	Transcutaneous oxygen
DRS	Diffuse reflectance spectroscopy
VO	Venous occlusion
FB	Fiber bundle
Mg	Magnesium
Ti	Titanium
*pO*_2_	Partial pressure of oxygen
LED	Light-emitting diode
MAP	Maximum amplitude projection
OR-PAM	Optical-resolution photoacoustic microscopy
PD	Photodiode
M	Mirror
FC	Fiber coupler/collimator
SMF	Single-mode optical fiber
P	Prism
GS	Galvanometer scanner
WT	Water tank
LS	Linear stage
RUT	Ring-shaped ultrasound transducer
OW	Optical window
PBM	Parabolic mirror
PBS	Phosphate-buffered saline
CBFv	Cerebral blood flow velocity
PAD	Peripheral artery disease
MSOT	Multispectral optoacoustic tomography
cSCC	Cutaneous squamous cell carcinoma
MVD	Microvascular density
DM	Distance metric
*MRO*_2_	Metabolic rate of oxygen
V-PDT	Vascular-targeted photodynamic therapy
a.u.	Arbitrary unit
PU	Perfusion unit
PW	Pulsed-wave
PDUS	Power Doppler ultrasound
PDX	Patient-derived xenograft
MVA	Microvascular area
MS-PAFI	Multispectral photoacoustic fluctuation imaging
TBI	Traumatic brain injury
SAH	Subarachnoid hemorrhage
VVF	Velocity-weighted volume of blood flow
AIA	Adjuvant-induced arthritis
DAS	Delay-and-sum
HFUS	High-frequency ultrasound
CBF	Cerebral blood flow
rBV	Relative blood volume
MROT	Magnetic resonance and optoacoustic tomography
OAT	Optoacoustic tomography
MRA	Magnetic resonance angiography
MMP	Matrix metalloproteinase
tMCAO	Transient middle cerebral artery occlusion
ICG	Indocyanine green
IPASC	International Photoacoustic Standardization Consortium
